# Host Identity and Urban Space Structure Phyllosphere Bacterial Communities Through Compositional Turnover in a Tropical Andean City

**DOI:** 10.3390/plants15203202

**Published:** 2026-10-19

**Authors:** Luisa María Múnera Porras, Santiago García Londoño, Héctor Alejandro Rodríguez Cabal, Miguel Pérez, Nancy J. Pino

**Affiliations:** 1 Research Group on Health and Sustainability, School of Microbiology, University of Antioquia, Calle 70 No. 52-21, Medellín 050010, Colombia; luisam.munera@udea.edu.co; 2 Diagnostic and Pollution Control Research Group (GDCON), School of Microbiology, University of Antioquia, Calle 70 No. 52-21, Medellín 050010, Colombia; santiago.garcia3@udea.edu.co; 3 Grupo de Biotecnología Vegetal, Facultad de Ciencias Agrarias, Universidad Nacional de Colombia Sede Medellín, Medellín 050034, Colombia; hearodriguezca@unal.edu.co; 4 Biosciences Research Group, Faculty of Health Sciences, University Institution Colegio Mayor de Antioquia, Medellín 050036, Colombia; miguel.perez@colmayor.edu.co

**Keywords:** epiphytic bacteria, phyllosphere, 16S rRNA, nestedness, turnover, urban ecology, community assembly, tropical Andes

## Abstract

Phyllosphere bacterial communities of urban vegetation remain poorly characterised in tropical Andean cities, where high year-round ultraviolet irradiance and compact valley topography differ from the temperate systems that dominate the literature. We characterised epiphytic bacterial communities on fifteen plants of three widely planted species: *Mangifera indica* L. (mango; Anacardiaceae), *Terminalia catappa* L. (tropical almond; Combretaceae) and *Thunbergia grandiflora* (Roxb. ex Rottler) Roxb. (blue trumpet vine; Acanthaceae), one per species in each of five urban zones of Medellín, Colombia, sampled in five surveys over nineteen months with three spatial subsamples per plant and survey (224 samples; 16S rRNA V3–V4). Host species and urban zone structured community composition across all five distance metrics examined (*R*^2^ = 0.19–0.30 and 0.34–0.37), yet neither affected any measure of alpha diversity. Partitioning beta diversity resolved why: host species and urban zone acted exclusively through taxon turnover, whereas survey acted exclusively through nestedness, driving a fivefold decline in richness that tracked a monotonic rise in relative humidity across the study period. The two spatial filters explained statistically independent fractions of variation (unique adjusted *R*^2^ = 0.236 and 0.174; shared fraction indistinguishable from zero). Bacterial communities from different parts of a single canopy differed by 69% of the dissimilarity separating host species, and this within-plant coherence weakened as communities contracted. Deterministic host selection and stochastic temporal contraction therefore appear to operate concurrently through complementary mechanisms in this system.

## 1. Introduction

Urban vegetation is increasingly understood not merely as structural green infrastructure but as a living interface between plants, microorganisms and the atmosphere [1,2]. Leaf surfaces collectively constitute one of the largest microbial habitats on Earth, the phyllosphere [3,4], where bacteria colonise exposed surfaces and experience pronounced fluctuations in water availability, nutrient supply, ultraviolet radiation, temperature and atmospheric deposition [5]. Colonisation depends on the balance among dispersal, local growth, survival and extinction under highly dynamic conditions. At the leaf scale, cuticular chemistry, epicuticular waxes, stomatal density, trichomes, exudate composition, leaf age and surface water retention determine the availability of microsites and resources [6,7,8]; at broader scales, immigration from air, soil, neighbouring vegetation, rain splash and anthropogenic sources determines the pool of taxa available for assembly [9,10]. The communities that establish therefore reflect both host-associated selection and source pool dynamics.

Host plant identity has been repeatedly identified as a major determinant of phyllosphere community structure. Studies in temperate trees have shown that host species explain a substantial fraction of bacterial community variation and that this effect frequently interacts with site and sampling time [11,12], and work in a neotropical forest has linked phyllosphere composition to host plant functional traits [13], indicating that host effects are mediated by measurable leaf properties rather than by taxonomic identity alone. Crucially, host effects need not be expressed as differences in the amount of diversity a leaf supports: different plant species may sustain comparable richness while selecting for distinct bacterial lineages, producing compositional differentiation without altering total diversity. This distinction is rarely tested explicitly.

It can, however, be formalised. Dissimilarity between two communities arises from either of two sources: replacement of some taxa by others, termed turnover, or ordered differences in richness in which the poorer community is a subset of the richer one, termed nestedness [14]. The two correspond to different assembly processes. Turnover is the signature expected of deterministic, niche-based selection in which distinct environments recruit distinct taxa from a shared regional pool; nestedness is the signature expected of differential colonisation or extinction in which the realised community contracts and expands without changing its identity. Despite its established use in macroecology, this decomposition has seldom been applied to phyllosphere systems, where studies typically report total dissimilarity without resolving the mechanisms that generate it [15]. Applying it shifts the question from how much variation each factor explains to how each factor acts.

Temporal variation constitutes a second major axis of assembly. Because leaf surfaces are directly exposed to the atmosphere, their communities respond rapidly to changes in rainfall, humidity, temperature, leaf phenology, deposition and wash-off [16,17]. In tropical systems, this dynamic is not organised around the temperate seasonal cycle, and sampling time may instead operate as a pulsed filter that periodically reshapes the set of lineages able to persist on leaves, rather than as a directional seasonal gradient [18].

A third axis operates within individual plants. Leaf age, height, orientation, light exposure and proximity to the ground vary systematically within a canopy, and the communities they support are not equivalent: intra-individual variation has been documented for a single host species [19] and across multiple temperate tree species, where canopy position explained a measurable fraction of community variation once host identity was accounted for [20]. What remains unknown is whether the coherence of individual plants as microbial habitats is constant through time or whether it varies with the state of the community itself. Designs combining spatial subsampling within individuals with repeated sampling over time can address this directly [21,22].

Urban environments add a further layer of complexity. Cities generate spatial heterogeneity through built surfaces, traffic emissions, particulate matter, modified microclimates and variable green infrastructure, and urban phyllosphere studies have shown that atmospheric pollution, green infrastructure and land use composition shape bacterial diversity and composition on leaves [23]. Phyllosphere plant–microbe interactions are accordingly recognised as relevant to Anthropocene pressures, including urbanisation, air pollution and climate stress [24].

Tropical urban phyllospheres nevertheless remain comparatively little studied. Research effort has been concentrated in temperate North America, Europe and East Asia, and tropical work has been conducted predominantly in natural forest rather than urban settings, a distribution that reflects the geography of sequencing infrastructure and research funding rather than any property of the systems themselves. Its consequence is that the baseline expectations of the field derive from ecosystems whose seasonal and radiative regimes differ substantially from those of tropical montane cities, where equatorial latitude sustains high ultraviolet irradiance throughout the year without seasonal modulation, narrow valley topography constrains ventilation, and the absence of marked thermal seasonality decouples temporal dynamics from the annual cycles that organise temperate studies.

Here, we characterise epiphytic phyllosphere bacterial communities of fifteen plants of three widely planted species, one per species in each of five urban zones of a tropical Andean city, sampled in five surveys over nineteen months with three spatial subsamples per plant and survey. We tested three hypotheses: that host identity and urban space act as statistically independent filters on community composition rather than as redundant sources of variation; that host-associated structuring is expressed through compositional turnover rather than through changes in diversity magnitude, whereas temporal variation acts principally on the amount of diversity a leaf supports; and that the coherence of individual plants as microbial habitats is not constant but varies with the state of the community, weakening as the realised community contracts.

## 2. Results

### 2.1. Sequencing Output and Construction of the Analytical Dataset

Amplicon sequencing of the V3–V4 region of the 16S rRNA gene from 224 phyllosphere samples yielded 168,120 amplicon sequence variants (ASVs). One sample from *T. catappa* (Este zone, survey M4) was not recovered after sequencing, so the final dataset comprised 224 of the 225 samples anticipated by the design of 15 plants × 5 surveys × 3 spatial subsamples. Survey dates and the meteorological conditions recorded at each sampling point are given in Table 1.

Chloroplast and mitochondrial sequences (6362 ASVs) were removed prior to phylogenetic reconstruction. Retaining only ASVs assigned to the domain Bacteria further excluded Archaea (39), Eukaryota (1) and features unassigned at domain level (53), yielding 161,665 bacterial ASVs and 44,325,146 reads. Taxonomic assignment was well resolved: 99.44% of reads were classified to phylum, 96.92% to family and 84.08% to genus, corresponding to 98.3%, 92.8% and 81.0% of individual ASVs respectively (Table S1).

The bacterial ASV table was strongly dominated by extremely rare features: 130,244 ASVs (80.6% of all bacterial ASVs) were detected in a single sample yet together accounted for only 2.52% of bacterial reads. This distribution is characteristic of residual denoising noise rather than of biologically meaningful rare taxa. An ecological filter retaining ASVs present in at least two samples and represented by at least ten reads therefore removed 81.5% of ASVs while preserving 97.46% of reads, yielding 29,894 ASVs and 43,197,788 reads. This filtered table underpins all analyses reported below, ensuring that alpha and beta diversity were estimated from identical underlying data.

Sequencing depth in the filtered table ranged from 49,216 to 1,043,162 reads per sample (median 153,048; Table S2). Rarefaction curves approached saturation well below 45,000 reads in all samples (Figure S1), and alpha diversity was therefore computed after rarefaction to this depth, which retained every sample.

### 2.2. Technical Assessment of the Sampling Survey Factor

Because each survey corresponds to an independent round of DNA extraction, library preparation and sequencing, the survey factor confounds ecological time with technical batch throughout this study. The diagnostic analyses in this section quantify that confounding; all subsequent statements about temporal variation should be read subject to it (Figure S2).

Median sequencing depth declined monotonically across surveys, from 342,529 reads in M1 to 105,891 in M4. Survey M1 contained 4667 survey-exclusive ASVs, an order of magnitude more than any other survey (66–499), a pattern consistent with its greater sequencing depth (Table S4).

A total of 12 of the 44 samples in survey M4 yielded fewer than 100 observed ASVs after rarefaction to 45,000 reads (range 20–79), whereas no sample in any other survey fell below this threshold. These twelve samples were distributed across all three host species (*T. catappa* *n* = 5, *T. grandiflora* *n* = 4, *M. indica* *n* = 3) and four of the five urban zones (Sur *n* = 5, Este *n* = 3, Norte *n* = 2, Oeste *n* = 2), indicating that the pattern was not restricted to a particular host or site. In every case the community was dominated by a single ASV at 18.2–96.4% relative abundance belonging to genera of the order Enterobacterales: *Serratia* (Yersiniaceae; three samples, up to 53.7%), *Pantoea* (Erwiniaceae; two samples, up to 96.4%), *Enterobacter* and *Kosakonia* (Enterobacteriaceae), together with five samples whose dominant ASV was unresolved at genus level. Consistently, the median relative abundance of the most abundant ASV per sample was 23.4% in M4 compared with 6.2% in M1 (Table S4).

Sequencing depth correlated with the second axis of the principal coordinates ordination (Spearman ρ = −0.427, *p* = 3.4 × 10^−11^) and weakly with the first (ρ = −0.163, *p* = 0.014). Critically, these correlations were unchanged after rarefaction (ρ = −0.427 and −0.153; Table S5), demonstrating that they do not arise from unequal sequencing effort but reflect the confounding of sequencing depth with survey.

Part of the diversity and compositional signal attributed to survey M4 is therefore of technical rather than ecological origin. All analyses were repeated excluding these twelve samples (Section 2.10), and the survey effect is interpreted throughout as an upper bound on temporal ecological variation.

### 2.3. Taxonomic Composition of the Urban Phyllosphere

**Figure 1:**
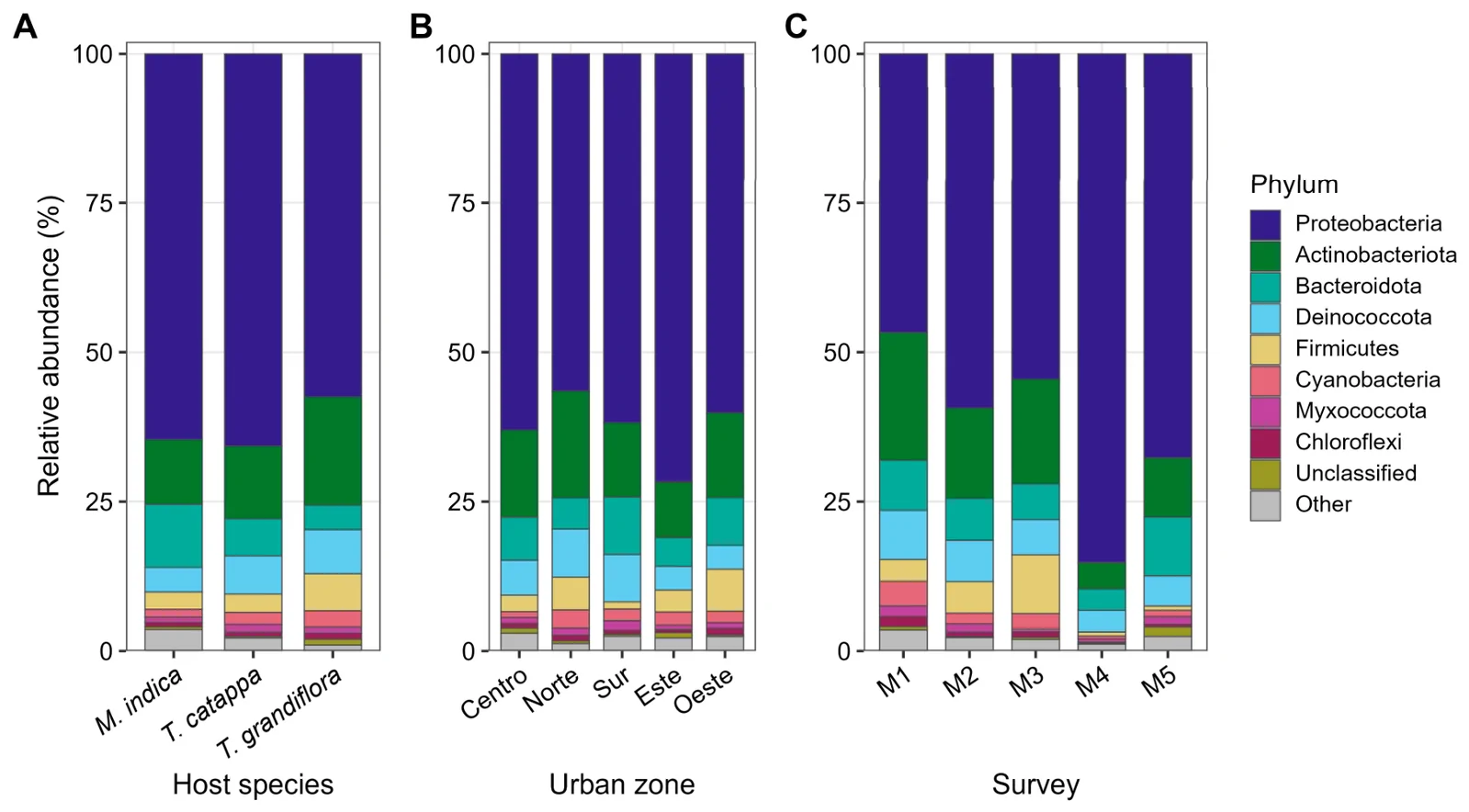
Phylum-level composition of epiphytic phyllosphere bacterial communities by (**A**) host species, (**B**) urban zone and (**C**) sampling survey, each survey being a single collection day on which all fifteen plants were sampled. Bars show mean relative abundances calculated within each sample and then averaged across the samples of each group so that every sample contributes equally regardless of sequencing depth. The nine most abundant phyla across the entire dataset were retained, and all remaining taxa pooled as “Other”; taxa were selected once globally so that colours denote the same phylum in all three panels. Phylum nomenclature follows SILVA 138. Based on 224 samples and 29,894 ASVs after ecological filtering.

The epiphytic phyllosphere was dominated by Proteobacteria (62.60% of mean relative abundance), followed by Actinobacteriota (13.68%), Bacteroidota (6.98%), Deinococcota (5.96%), Firmicutes (4.03%) and Cyanobacteria (2.01%); all remaining phyla accounted for less than 1.2% each (Figure 1). Phylum nomenclature follows SILVA 138; *Pseudomonadota* and *Bacillota* are the validly published names of Proteobacteria and Firmicutes, respectively. Within Proteobacteria, Gammaproteobacteria (35.87%) exceeded Alphaproteobacteria (26.73%). Cyanobacterial reads belonged to Cyanobacteriales (1.97%) and contained no residual chloroplast sequences, confirming the effectiveness of the non-target filter.

At the family level, Beijerinckiaceae was the most abundant lineage (14.12%), followed by Erwiniaceae (12.02%), Enterobacteriaceae (11.35%), Deinococcaceae (5.66%), Acetobacteraceae (5.43%), Hymenobacteraceae (4.65%) and Sphingomonadaceae (3.99%). The dominant genera were *Pantoea* (10.19%) and *Methylobacterium*–*Methylorubrum* (10.00%), followed by *Deinococcus* (5.66%), *Hymenobacter* (4.62%), *Sphingomonas* (3.20%), an unresolved Acetobacteraceae lineage designated 1174-901-12 in SILVA (3.04%), *Enterobacter* (2.92%), *Massilia* (2.68%), *Serratia* (2.52%) and *Pseudomonas* (2.12%) (Table S6). Composition at the family and genus level is shown by host species in Figure S3 and by survey in Figure S4.

Two features of this composition warrant explicit qualification. First, the combined relative abundance of *Deinococcus* and *Hymenobacter*, two carotenoid-pigmented lineages associated with tolerance to ultraviolet radiation and desiccation, reached 10.28%. Second, Enterobacteriaceae ranked third at the family level but was not a structural component of this system: its median relative abundance was 14.69% in survey M4 (mean 36.24%) compared with 0.04–0.81% in the remaining surveys (Table S6). This family is therefore reported descriptively and excluded from ecological interpretation.

### 2.4. A Hierarchy of Community Variation from Within-Plant to Between-Species

**Figure 2:**
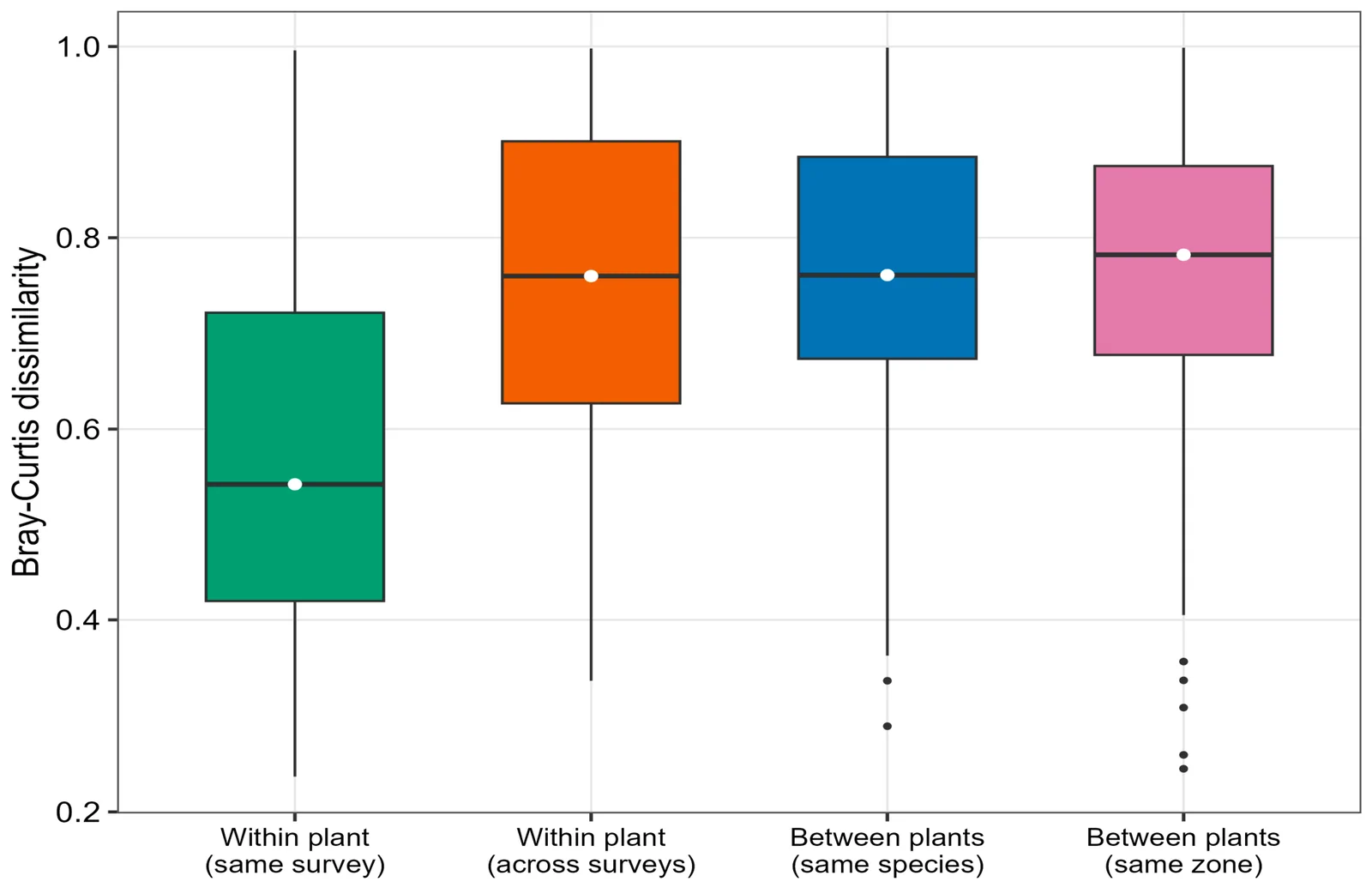
Bray–Curtis dissimilarity across four nested scales of the sampling design. From left to right: between spatial subsamples of the same plant collected in the same survey (n = 223 pairs); between subsamples of the same plant collected in different surveys (n = 1338); between plants of the same host species in different urban zones within a survey (n = 1338); and between plants of different host species in the same urban zone within a survey (n = 669). Each survey was a single collection day on which all fifteen plants were sampled. Boxes show the median and interquartile range and whiskers extend to 1.5 times the interquartile range. Median dissimilarity within a single canopy on a single day (0.542) represented 69% of that observed between plants of different host species (0.782).

Community dissimilarity increased across four nested scales of the sampling design (Figure 2). Subsamples collected from different parts of the same canopy on the same day differed by a median Bray–Curtis dissimilarity of 0.542 (n = 223 pairs). Dissimilarity rose to 0.760 between subsamples of the same plant in different surveys (n = 1338), to 0.761 between plants of the same host species in different urban zones within a survey (n = 1338), and to 0.782 between plants of different host species in the same zone within a survey (n = 669); all remaining comparisons averaged 0.854 (Table 2).

Within-plant heterogeneity therefore represented 69% of the dissimilarity observed between plants of different host species. Moving from one plant to another of a different species in the same zone added only 0.240 dissimilarity units beyond what already separated two parts of a single canopy.

Under matched conditions of surveys, the median distance from a given sample to plants of a different host species in the same zone (0.772) did not differ from the distance to plants of the same species in other zones (0.755; Wilcoxon signed-rank, *p* = 0.29), indicating that relocating a host species across the city alters its phyllosphere community to a comparable extent as changing the host species at a fixed location. Aitchison distances gave equivalent results (Figure S5).

### 2.5. Sampling Survey, but Neither Host Identity nor Urban Zone, Structures Alpha Diversity

**Figure 3:**
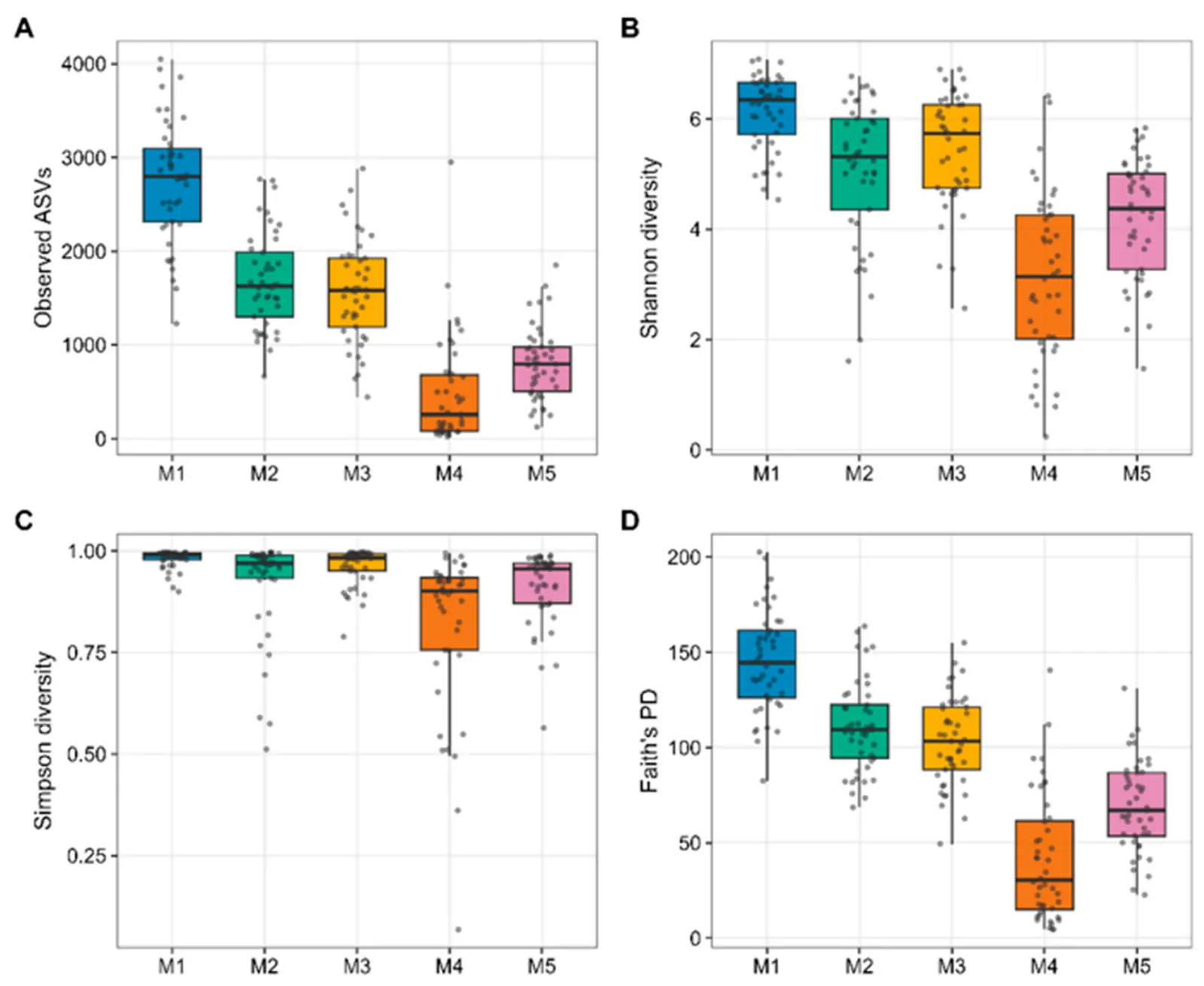
Alpha diversity of epiphytic phyllosphere bacterial communities across the five sampling surveys: (**A**) observed ASV richness, (**B**) Shannon diversity, (**C**) Simpson diversity and (**D**) Faith’s phylogenetic diversity. Each survey was a single collection day on which all fifteen plants were sampled. Metrics were computed on the ecologically filtered table rarefied to 45,000 reads per sample. Boxes show the median and interquartile range, whiskers extend to 1.5 times the interquartile range, and points represent individual samples (n = 224). Letters above each box denote groups that do not differ significantly in post hoc contrasts from the linear mixed-effects model fitted at the plant–survey level (n = 75), with *p*-values adjusted by the Benjamini–Hochberg procedure. Survey was significant for every metric (observed ASV richness *F*_4_,_56_ = 63.73, *p* = 3.4 × 10^−20^).

Alpha diversity was analysed at the plant–survey level (n = 75), the level at which the survey factor varies (Figure 3; Table 3).

Survey explained substantial variation in every metric (*F*_4_,_56_ = 63.73, *p* = 3.4 × 10^−20^ for observed ASV richness; all six metrics significant at *p* < 10^−5^, Table 3). Mean observed richness declined more than fivefold, from 2757.9 ± 641.7 ASVs in M1 to 477.1 ± 562.9 in M4, with intermediate values in M2, M3 and M5, and equivalent gradients were obtained for Chao1, Shannon, inverse Simpson and Faith’s phylogenetic diversity (Table S7). Post hoc contrasts ordered surveys as M1 > M2 ≈ M3 > M5 > M4, with only M2 and M3 failing to differ; all remaining pairwise contrasts were significant after Benjamini–Hochberg correction (Table S8).

Observed richness was inversely related to mean relative humidity across surveys (Spearman ρ = −0.90, *p* = 0.037), whereas mean air temperature per survey, which ranged from 21.6 to 23.5 °C, showed no significant association (ρ = 0.60, *p* = 0.28). Because relative humidity increased monotonically with survey order, this association is collinear with both the temporal sequence and the sequencing batch; it is interpreted in Section 3.

Importantly, the survey effect was stronger on the ecologically filtered table than on the unfiltered one (η^2^ = 0.691 versus 0.611 for observed richness), demonstrating that it is not an artefact of low-abundance sequencing noise (Figure S6; Table S7).

Host species had no detectable effect on any alpha diversity metric, either at the plant–survey level (*p* = 0.22–0.84) or at the plant level (n = 15; *p* = 0.17–0.84), and accounted for less than 0.5% of the marginal variance in every index (η^2^ ≤ 0.003). Evidence for an effect of urban zone was weak and inconsistent: of the six metrics tested, only Simpson diversity reached nominal significance (*p* = 0.029 at plant–survey level; *p* = 0.052 at plant level), with Shannon and Faith’s phylogenetic diversity marginal (*p* = 0.055 and 0.083) and the remaining metrics far from significance (*p* ≥ 0.51) (Figure S7; Table S7). We therefore do not interpret urban zone as a determinant of alpha diversity in this system.

Random intercept variance for plant identity was small but non-zero for richness-based metrics (intraclass correlation 0.058 for observed richness, 0.062 for Chao1 and 0.025 for Faith’s phylogenetic diversity) and zero for evenness-weighted metrics. Denominator degrees of freedom for the between-plant factors were 8, as expected from a design comprising fifteen plants (Table 3).

### 2.6. Host Identity and Urban Zone Structure Community Composition Independently

**Figure 4:**
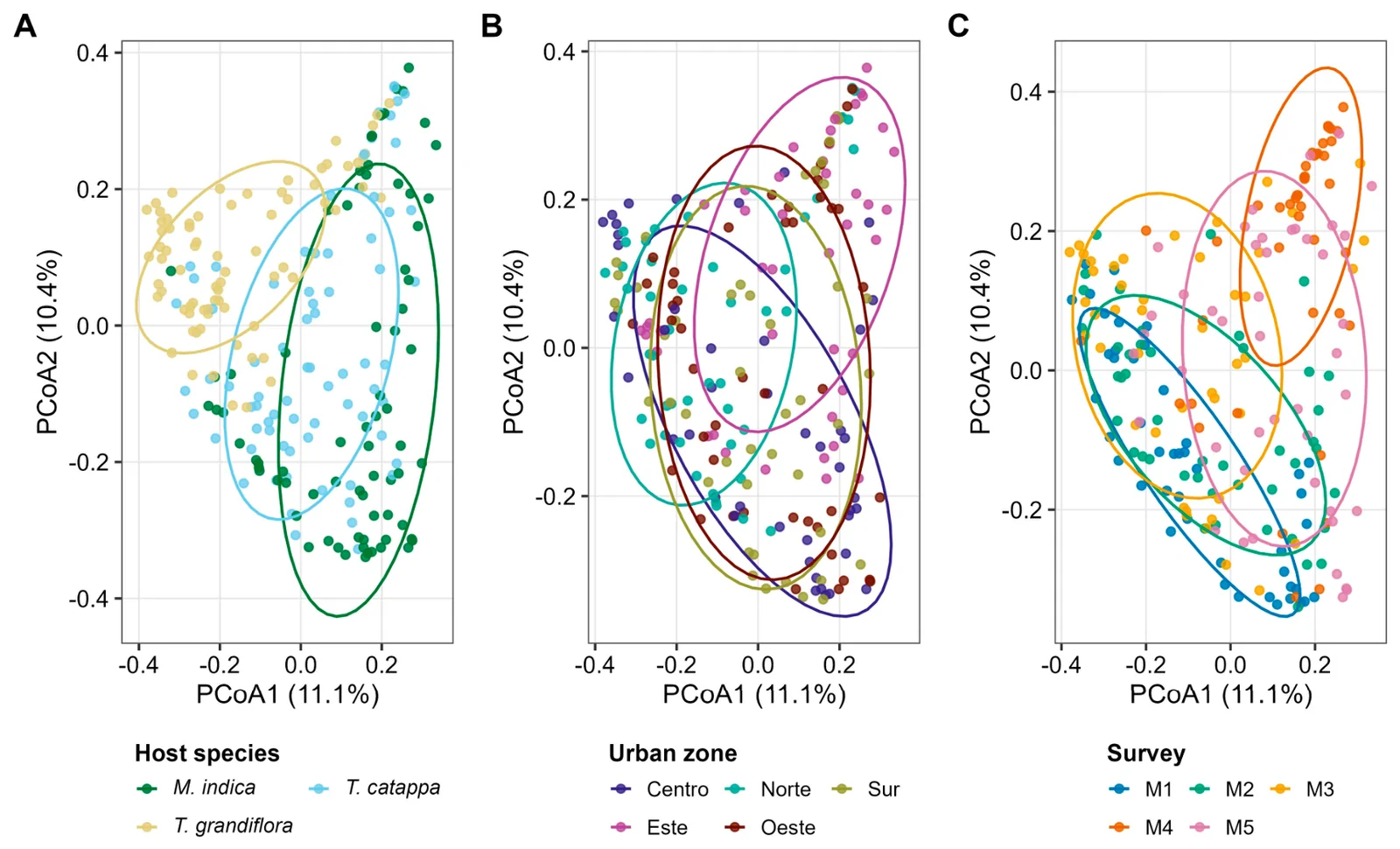
Principal coordinates analysis of Bray–Curtis dissimilarities among 224 phyllosphere samples, with points coloured by (**A**) host species, (**B**) urban zone and (**C**) sampling survey, each survey being a single collection day on which all fifteen plants were sampled. Ellipses enclose 68% of samples per group and axis labels give the percentage of total variation explained by each coordinate. This ordination is used for visualisation only: all tests of group separation are permutational and operate on the full distance matrix rather than on a reduced-dimensional projection. Statistical tests of host species and urban zone were performed on plant-level community profiles (n = 15) and tests of survey on individual samples with permutations restricted within plants, as reported in Table 4. A projection including the third principal coordinate is given in Figure S8.

**Figure 5:**
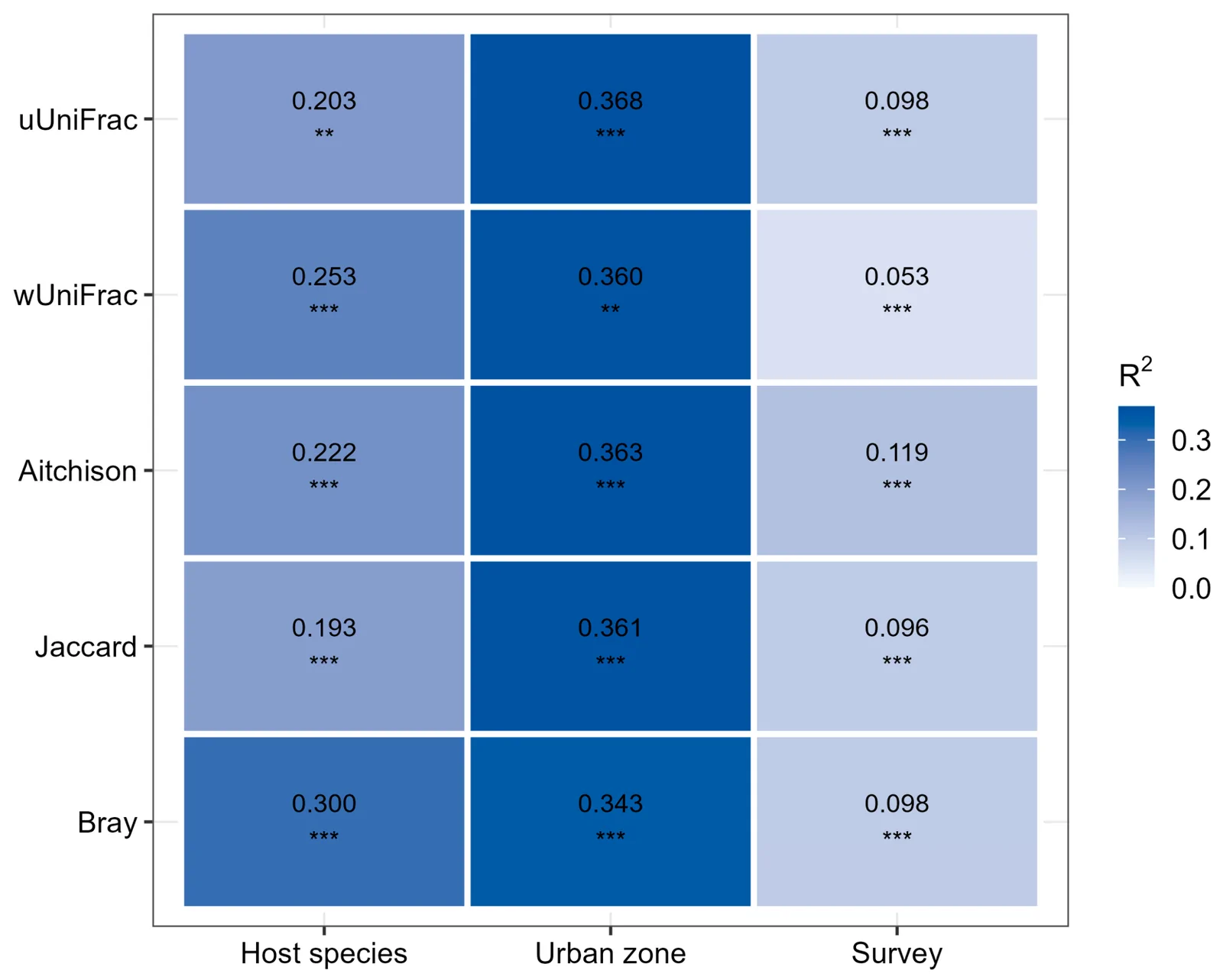
Effect sizes of the three ecological factors on community composition across five distance metrics. Cell values give the marginal PERMANOVA coefficient of determination (*R*^2^) and asterisks the significance level (*** *p* < 0.001; ** *p* < 0.01). Because the three factors vary at different levels of the design, each was tested at the level at which it varies: host species and urban zone on plant-level community profiles obtained by summing counts within each plant and rarefying to the minimum plant-level depth (n = 15; 9999 permutations), and survey—each survey being a single collection day on which all fifteen plants were sampled—on the 224 individual samples with permutations restricted within plants. Coefficients are comparable among metrics within a factor but not directly comparable in absolute magnitude between the two levels of analysis. Urban zone consumes four degrees of freedom against two for host species, which inflates its unadjusted *R*^2^; the degree-of-freedom-adjusted comparison is given in Figure 6.

Community composition was visualised by principal coordinates analysis of Bray–Curtis dissimilarities (Figure 4; a projection including the third coordinate is given in Figure S8). Because host species and urban zone vary between plants, their effects were tested on plant-level community profiles (n = 15), whereas survey, which varies within plants, was tested on individual samples with permutations restricted within plants (Figure 5; Table 4).

Both spatial factors were significant across all five distance metrics (Table 4). Host species explained between 19.3% and 30.1% of community variation among plants, the largest effect being obtained with Bray–Curtis dissimilarity (R^2^ = 0.301, F = 3.37) and the smallest with Jaccard (R^2^ = 0.193, F = 1.73; all *p* ≤ 1.9 × 10^−3^). Urban zone explained between 34.3% and 36.8%, a narrower range across metrics (all *p* ≤ 1.5 × 10^−3^).

**Figure 6:**
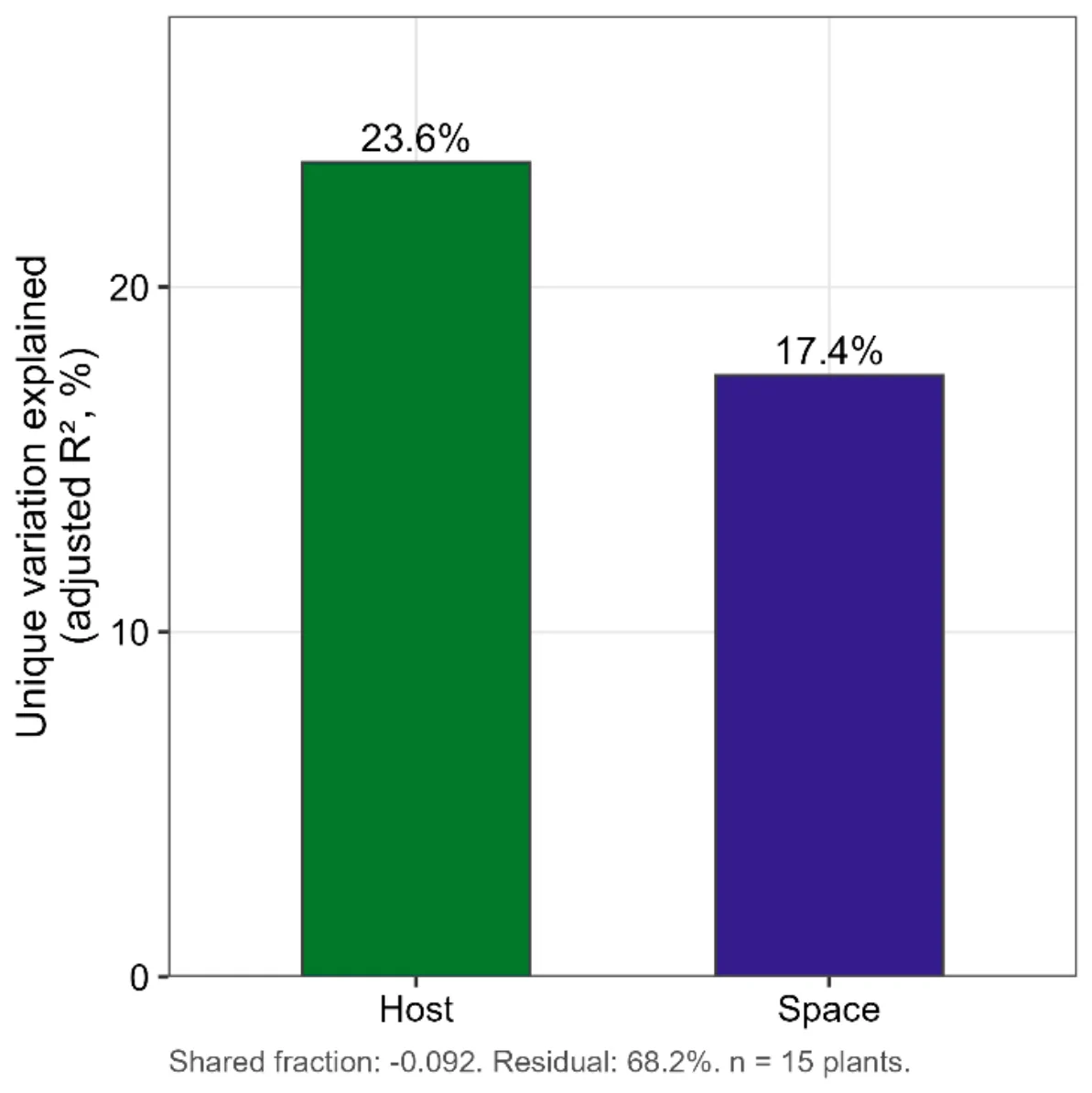
Unique fractions of community variation attributable to host species and urban zone, estimated by variance partitioning of Hellinger-transformed plant-level community profiles (n = 15 plants). Unique fractions were obtained by partial redundancy analysis with the remaining factor included as a conditioning variable and are expressed as adjusted *R*^2^, which corrects for the number of degrees of freedom consumed by each term. Host species accounted for 23.6% and urban zone for 17.4% of adjusted variation. The joint fraction shared by the two factors was indistinguishable from zero (adjusted *R*^2^ = −0.092), confirming their statistical independence in this orthogonal design; 68.2% of variation remained unexplained.

These unadjusted coefficients are not directly comparable between the two factors because urban zone consumes four degrees of freedom against two for host species. Variance partitioning on plant-level Hellinger-transformed profiles, which adjusts for model complexity, attributed 23.6% of adjusted variation uniquely to host species and 17.4% uniquely to urban zone, reversing the apparent ranking (Figure 6; Table 5). The joint fraction was indistinguishable from zero (adjusted R^2^ = −0.092), confirming that the two factors were statistically independent by design, and 68.2% of variation remained unexplained.

Survey structured composition across all five metrics under permutations restricted within plant (R^2^ = 0.053–0.119, all *p* = 1 × 10^−4^; Table 4).

Multivariate dispersion did not differ significantly among host species (F = 0.16, *p* = 0.86) or among urban zones (F = 0.36, *p* = 0.83) at plant level. Because these tests are based on five and three plants per group respectively, they have limited power, and this result constitutes absence of evidence for dispersion heterogeneity rather than evidence of homogeneity. Survey, by contrast, showed strong dispersion heterogeneity in every metric (F up to 97.2, all *p* = 1 × 10^−4^; Figure S9; Table S9), so survey effects reflect both displacement of centroids and pronounced differences in within-group variability.

### 2.7. Host and Spatial Filters Act Through Turnover; Campaign Acts Through Nestedness

**Figure 7:**
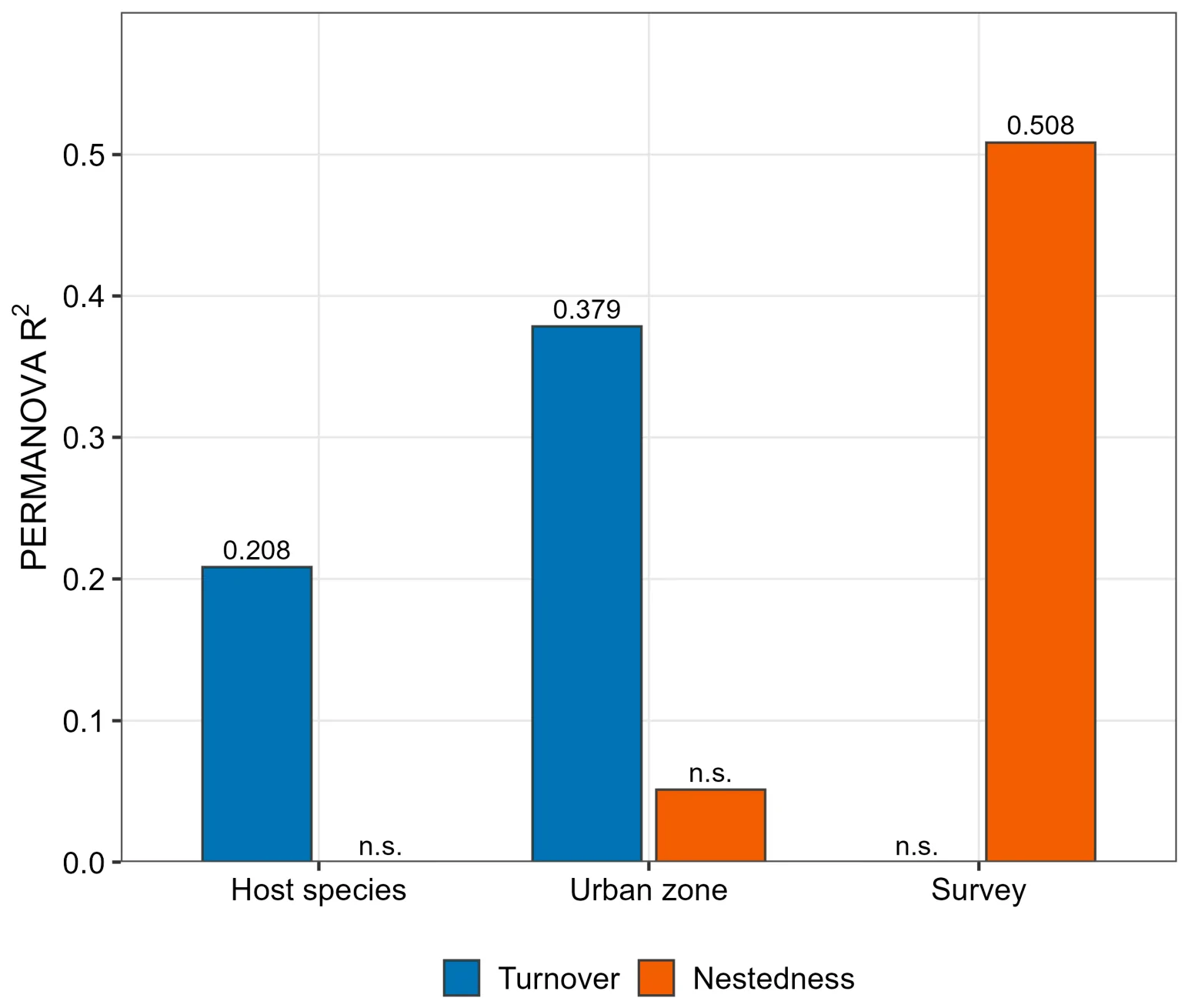
Partitioning of Jaccard dissimilarity into its turnover and nestedness components for each ecological factor. Bars give marginal PERMANOVA coefficients of determination for each component analysed separately. Host species and urban zone were tested on plant-level rarefied profiles (n = 15) and survey on individual samples with permutations restricted within plants. Host species and urban zone structured only the turnover component (*R*^2^ = 0.208 and 0.379; both *p* ≤ 2 × 10^−4^) with no detectable effect on nestedness, whereas survey structured only the nestedness component (*R*^2^ = 0.508, *p* = 1 × 10^−4^) with no detectable effect on turnover. Because the nestedness component of Jaccard dissimilarity is not a metric distance, permutational tests on this matrix can return negative sums of squares; such cases are shown as zero and labelled n.s., and the comparison of component means reported in Table 6 provides the primary evidence for these conclusions.

Partitioning Jaccard dissimilarity into turnover and nestedness components revealed a consistent dissociation of mechanisms among the three factors (Figure 7; Table 6).

At the plant level, host species and urban zone structured the turnover component (host species *R*^2^ = 0.208, *F* = 2.02, *p* = 2 × 10^−4^; urban zone *R*^2^ = 0.379, *F* = 1.83, *p* = 1 × 10^−4^) but had no detectable effect on nestedness (*p* = 0.92 and 0.87). Component means confirmed this pattern independently of the permutational tests: between host species, mean turnover increased relative to within-species comparisons (0.663 to 0.699) while mean nestedness decreased (0.043 to 0.035); urban zone showed the same pattern (turnover 0.642 to 0.697; nestedness 0.044 to 0.036).

Survey showed the opposite. Under permutations restricted within plants, survey structured the nestedness component strongly (*R*^2^ = 0.508, *F* = 56.61, *p* = 1 × 10^−4^) with no detectable effect on turnover (*p* = 1.0).

Host identity and urban space therefore appear to influence community composition by replacing taxa, whereas temporal variation alters the realised community by contracting and expanding it without changing its identity.

### 2.8. Temporal Variation Fluctuates Rather than Accumulating Directionally

Community dissimilarity between subsamples of the same plant in different surveys increased weakly with elapsed time (Spearman ρ = 0.190, *p* = 2.2 × 10^−12^, n = 1338 pairs; Figure S10), but the relationship was not monotonic. All four intervals involving survey M4 exceeded a median Bray–Curtis dissimilarity of 0.89 (M4–M5, 131 days, 0.890; M3–M4, 231 days, 0.932; M2–M4, 363 days, 0.932; M1–M4, 434 days, 0.946), whereas none of the six remaining intervals did (range 0.605–0.789). Notably, the longest interval in the study, M1 to M5 (565 days), yielded a lower median dissimilarity (0.732) than the 231-day M3–M4 interval (Table S10).

Temporal turnover in this system therefore does not accumulate progressively with elapsed time; the weak positive correlation is driven by the compositional distinctiveness of survey M4 rather than by directional community change.

### 2.9. Within-Plant Community Coherence Weakens as Communities Contract

**Figure 8:**
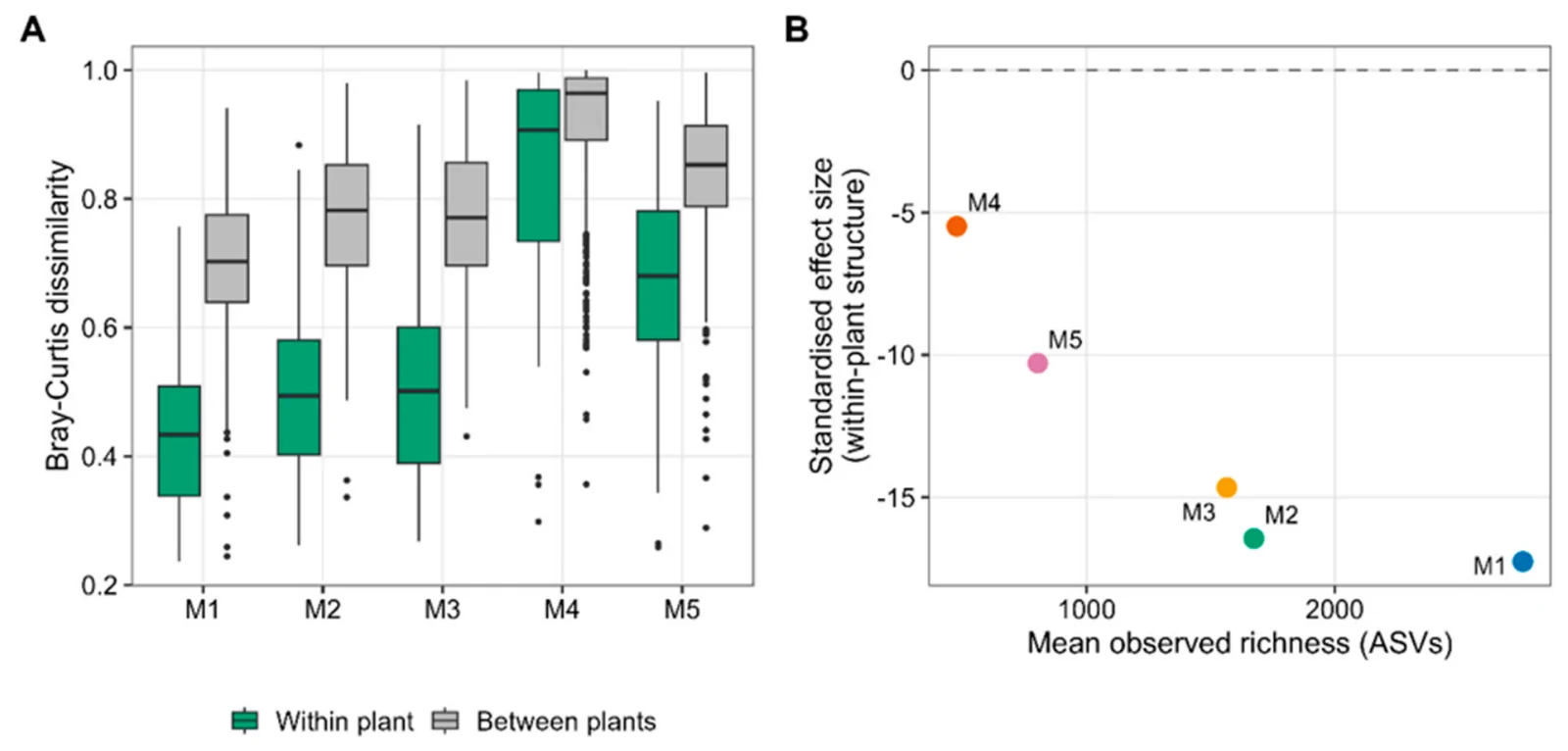
Within-plant community heterogeneity and its relationship with community richness. (**A**) Bray–Curtis dissimilarity between spatial subsamples of the same plant (green) and between different plants (grey) within each sampling survey, each survey being a single collection day on which all fifteen plants were sampled. (**B**) Standardised effect size of within-plant community structure against mean observed richness for each survey. The standardised effect size derives from a null model in which subsamples were randomly reassigned to plants within each survey (999 randomisations), preserving the observed richness of every sample; increasingly negative values indicate that subsamples of the same plant are more similar to one another than expected by chance. Within-plant structure was significant in every survey (all *p* = 0.001) but substantially stronger in the three high-richness surveys (M1, M2, M3) than in the two low-richness ones (M4, M5). Equivalent results were obtained using the ratio of within-plant to between-plant dissimilarity, which cancels the mechanical dependence of Bray–Curtis dissimilarity on richness, and after excluding the twelve low-richness samples of survey M4.

Within-plant heterogeneity varied markedly among surveys, from a median Bray–Curtis dissimilarity of 0.434 in M1 to 0.907 in M4, and was inversely related to community richness across surveys (Spearman ρ = −1.00, *p* = 0.017; Figure 8) and positively related to mean relative humidity (ρ = +0.90, *p* = 0.037). By contrast, within-plant heterogeneity did not differ among host species (*M. indica* 0.539, *T. catappa* 0.535, *T. grandiflora* 0.574; Kruskal–Wallis χ^2^ = 0.30, *p* = 0.86).

Because Bray–Curtis dissimilarity increases mechanically as richness declines, three independent controls were applied (Table S11). First, the ratio of within-plant to between-plant dissimilarity within each survey, which cancels the mechanical inflation shared by both terms, showed the same relationship with richness, rising from 0.617 in M1 to 0.941 in M4 (ρ = −1.00, *p* = 0.017). Second, excluding the twelve low-richness samples of survey M4 reduced its within-plant dissimilarity from 0.907 to 0.755 while leaving all other surveys unchanged and preserving the rank ordering. Third, a null model randomly reassigning subsamples to plants within each survey showed that subsamples of the same plant were more similar than expected by chance in every survey (all *p* = 0.001), but that the magnitude of this effect was markedly greater in the three high-richness surveys (standardised effect sizes −17.3, −16.5 and −14.7 for M1, M2 and M3) than in the two low-richness ones (−10.3 for M5 and −5.5 for M4). Repeating the null model after excluding the low-richness samples reproduced this contrast (−17.3, −16.5, −14.7, −10.1 and −6.5 respectively), with the null expectation for M4 falling from 0.961 to 0.914 and therefore well below the dissimilarity ceiling.

Individual plants were therefore detectably coherent as microbial habitats throughout the study, but this coherence eroded as communities contracted: as richness declined, different parts of the same canopy retained increasingly divergent subsets of the community.

### 2.10. Sensitivity Analyses

Excluding the twelve low-richness samples of survey M4 did not alter any qualitative conclusion. The survey effect on alpha diversity remained overwhelming (observed richness *F*_4_ = 104.48, *p* = 2.1 × 10^−44^), the absence of a host effect on alpha diversity was unchanged (*p* = 0.22–0.53), and at sample level the ranking of compositional effect sizes shifted in favour of host species (*R*^2^ = 0.096) ahead of urban zone (0.095) and survey (0.092; all *p* = 1 × 10^−4^) (Table S12).

## 3. Discussion

The central finding of this study is not that host identity, urban space and sampling time each structure phyllosphere bacterial communities—a result consistent with previous work in temperate trees [11,20]—but that in this system they do so through mechanistically distinct pathways. Decomposing beta diversity into its turnover and nestedness components [14] revealed a consistent dissociation: host identity and urban zone acted exclusively through taxon replacement, whereas survey acted exclusively through ordered differences in richness. Whether this dissociation is a general property of phyllosphere assembly or specific to the conditions examined here cannot be determined from a single study system, and we offer it as a hypothesis for testing in other settings.

This distinction matters because the two components correspond to different fates for individual lineages. Turnover implies substitution: a taxon present in one community is absent from another, and a different taxon takes its place. It is the signature expected of deterministic, niche-based selection, in which distinct environments recruit distinct organisms from a shared regional pool, and it implies that the pool itself is not limiting—there are always lineages available to occupy the habitat, and which ones do so depends on the match between organism and environment. Nestedness implies subsetting: the poorer community contains a fraction of what the richer one contains, with no compensating gains. It is the signature expected of differential colonisation or extinction, in which the same pool is sampled more or less completely, and it implies that the constraint operates on the number of lineages that can establish or persist rather than on their identity.

Read together with the alpha diversity results, host identity and urban zone determined which bacteria were present without altering how many, whereas survey determined how many without substantially altering which. The two spatial filters therefore behaved as selective filters and the temporal filter as a bottleneck. This distinction is invisible to analyses that report total dissimilarity alone, which would register all three factors as significant and of comparable magnitude, as indeed our own undecomposed PERMANOVA does, while concealing that they act on different properties of the community. Had the pattern been reversed, with host identity generating nestedness, the interpretation would have been quite different: it would have implied that some hosts are simply poorer habitats than others, supporting impoverished subsets of the same assemblage rather than distinct assemblages. That is not what we observe, and the distinction bears directly on how host effects in the phyllosphere should be understood. In systems where a single dominant driver of phyllosphere structure is reported, processes acting on these two distinct properties of the community may be difficult to separate unless the decomposition is applied explicitly.

Turning first to the host filter, host species structured community composition consistently across five distance metrics spanning abundance-weighted, presence–absence, compositional and phylogenetic approaches, and accounted for the largest unique fraction of adjusted variation among plant-level profiles. Yet it had no detectable effect on any measure of alpha diversity at any level of analysis. Mean observed richness differed by less than 8% among the three hosts, and host identity accounted for under 0.5% of the marginal variance in every diversity index. This pattern argues against interpreting host effects as a gradient of habitat harshness, under which a less hospitable leaf surface would support a species-poor subset of the community found on a more hospitable one. It is instead consistent with each host presenting a distinct but comparably permissive habitat. Leaf surfaces differ among species in cuticular wax composition, trichome density and morphology, stomatal density, surface topography, water retention and the composition of leachates and exudates [6,7,8], and it is these qualitative differences, rather than a quantitative gradient of habitability, that our results implicate.

Two caveats temper this interpretation. First, *T. grandiflora* is a climbing vine whereas *M. indica* and *T. catappa* are trees, so host identity is partially confounded with growth form and, consequently, with canopy position, sampling height, proximity to the ground and exposure to rain splash. Second, three host species cannot separate phylogenetic distance from functional trait differences; studies incorporating leaf functional traits directly [13] would be required to identify which specific traits mediate the selection observed. The absence of a host effect on alpha diversity should accordingly be read in conjunction with its negligible effect size rather than as demonstrated equivalence.

A structural constraint of the design limits how far these results generalise. Because one plant of each species was monitored in each zone, individual plant identity is fully confounded with the species × zone combination: what we report as a host species effect is the effect of three particular individuals per species, and what we report as a zone effect is the effect of three particular individuals per zone. Interactions between the two factors are correspondingly not estimable. Consistency across five distance metrics and across five surveys argues against the pattern being driven by the idiosyncrasy of any single plant, and the plant-level random intercept in the alpha diversity models accounted for a small but non-zero share of variance in richness-based metrics (intraclass correlation 0.025–0.062), indicating that between-plant variation was modest relative to variation among surveys. These are nevertheless indirect arguments. Establishing that the effects reported here are properties of the species and of the zones rather than of the fifteen individuals sampled would require several plants of each species within each zone, and we regard the present results as a hypothesis-generating baseline for such a design rather than as a demonstration of species-level generality.

Urban zone structured community composition across all five distance metrics and, like host identity, acted through turnover rather than nestedness. This is notable given the extent involved: the five zones lie within a single metropolitan area in a narrow Andean valley, separated by a few kilometres, and were selected to span the cardinal sectors of the city rather than to contrast land use, traffic density or vegetation cover, which are broadly comparable among them. The spatial signal we detect is therefore not attributable to the gross environmental contrasts that urban phyllosphere studies have most often examined [23].

Two candidate explanations remain, and the present design cannot discriminate between them. The first is microclimate. Although similar in land use, the zones differ consistently in atmospheric conditions: mean relative humidity spans 14 percentage points and mean air temperature 2.3 °C among them, with a ranking that was stable across all five surveys. Leaf surface water availability is a principal determinant of epiphytic bacterial survival and growth, so a persistent microclimatic gradient of this magnitude could plausibly select for different assemblages even in the absence of land use contrast. Consistent with this, the zone effect on alpha diversity was weak and inconsistent while the effect on composition was substantial: a microclimatic gradient that alters which taxa persist need not alter how many do. The second is dispersal. Position within the valley governs ventilation and the trajectories along which airborne colonists arrive, and distance alone can generate compositional differentiation through dispersal limitation and priority effects, without any difference in habitat quality. The relative weight of these processes cannot be resolved here.

That a spatial signal of this magnitude is detectable at all among sites of broadly similar urban character is, we suggest, the more informative result. The equivalence of the between-species and between-zone contrasts under matched conditions (median 0.772 and 0.755; *p* = 0.29) indicates that moving a given host species a few kilometres across the city changes its phyllosphere community about as much as changing the host species at a fixed location. Urban phyllosphere heterogeneity therefore appears to operate at scales considerably finer than the land use categories through which it is usually approached.

An apparent discrepancy between two of our analyses merits explanation. Unadjusted PERMANOVA coefficients were consistently larger for urban zone (*R*^2^ = 0.343–0.368) than for host species (0.193–0.301), yet variance partitioning attributed a larger unique fraction to host species (23.6% versus 17.4%). This reversal arises because urban zone consumes twice as many degrees of freedom, which inflates unadjusted *R*^2^, whereas variance partitioning corrects for model complexity. The adjusted values therefore provide a better comparison between the two filters, while the unadjusted coefficients remain informative for comparing metrics within a factor.

The temporal filter, by contrast, operated on a different property of the community altogether. Survey produced by far the greatest effect on alpha diversity, with observed richness declining more than fivefold from M1 to M4 and accounting for 69.1% of the marginal variance. This effect was stronger on the ecologically filtered dataset than on the unfiltered one, demonstrating that it is not an artefact of low-abundance denoising noise. That seasonal and climatic variation affects phyllosphere community diversity is well established, and the temporal effect reported here is not in itself unexpected. What the decomposition of beta diversity adds is the character of that effect: survey acted exclusively on the nestedness component, with no detectable contribution to turnover, whereas host identity and urban zone showed precisely the opposite. Communities sampled in different surveys were therefore not composed of different taxa; they were richer or poorer subsets drawn from a comparable pool—a distinction that is invisible to analyses reporting total dissimilarity alone.

The meteorological record offers a plausible environmental account of this pattern. The Aburrá Valley has a bimodal rainfall regime with wet seasons in April–May and September–November, and the five surveys sampled both wet and dry phases. Mean relative humidity nevertheless rose monotonically across the study rather than oscillating with the bimodal regime, from 58.0% in M1 to 77.9% in M5, and was inversely related to observed richness (ρ = −0.90, *p* = 0.037) and positively related to the erosion of within-plant coherence (ρ = +0.90, *p* = 0.037); mean air temperature, which varied over a narrower range, showed no significant association. Two non-exclusive mechanisms could link wetter conditions to reduced richness on leaf surfaces: rainfall-driven wash-off of epiphytic colonists, and competitive dominance by fast-growing copiotrophs under high leaf surface water availability—the latter consistent with the dominance of Enterobacterales observed in the low-richness samples of M4. That discrete environmental events can reconfigure the number and composition of taxa recovered from a microbial community has been documented in other systems; in thermophilic composting, for example, the mechanical turning of the pile partially restored the community profile observed at the start of the process, resetting rather than progressively transforming the assemblage [25]. The nestedness signature we observe is the phyllosphere analogue of such a resetting.

This account must be qualified in two respects. First, because relative humidity increased monotonically across the study period, it is collinear with survey order and, consequently, with sequencing batch. The association reported here is therefore offered as a plausible environmental account of the temporal pattern rather than as a demonstrated causal relationship; distinguishing these possibilities would require surveys distributed across humidity conditions independently of chronological order. Second, three observations indicate that the survey effect is not purely ecological. Median sequencing depth declined monotonically from M1 to M4; survey M1 contained an order of magnitude more survey-exclusive ASVs than any other survey; and twelve of the 44 samples in M4 yielded fewer than 100 ASVs while no sample in any other survey did, these being dominated by fast-growing Enterobacterales in a pattern consistent with proliferation of copiotrophic taxa at some point between collection and DNA extraction rather than with ecological contraction of the resident community, although the specific cause could not be determined. Because survey, extraction round, library preparation and sequencing run coincide exactly, no statistical adjustment can separate them within this design. What can be said is bounded: the magnitude of the temporal effect on richness is an upper bound rather than an estimate, and the mechanism through which it acts—nestedness rather than turnover—is the more robust of the two findings, since it concerns the relative contribution of two components of the same dissimilarity matrix and is therefore less sensitive to a uniform shift in detection sensitivity between batches. Notably, excluding the twelve low-richness samples shifted the ranking of sample-level effect sizes in favour of host identity, reinforcing the host effect as the more conservative finding.

Consistent with a fluctuating rather than a directional process, temporal change did not accumulate with elapsed time: community dissimilarity increased only weakly, and the relationship was not monotonic, with the longest interval in the study producing a lower median dissimilarity than a 231-day interval involving survey M4. Phyllosphere communities in this system therefore appear to fluctuate among states rather than to undergo succession, a pattern reported previously for phyllosphere systems subject to strong environmental forcing [16,17] and consistent with a model in which recurrent environmental windows repeatedly reset community richness.

Beneath these three filters lies a fourth scale of variation that our design was able to resolve. That phyllosphere communities vary within a single canopy is established: Laforest-Lapointe et al. [20] sampled six canopy positions in five temperate tree species and found that canopy location explained 6% of total community variation, rising to 22% of the residual variation once host species identity was accounted for, and Leff et al. [19] reported comparable within-canopy structure for *Ginkgo biloba*. Our estimate is consistent with this body of work: subsamples collected from different parts of the same canopy on the same day differed by a median Bray–Curtis dissimilarity of 0.542, equivalent to 69% of the dissimilarity separating plants of different host species. The leaf surface of a single individual therefore harbours community variation of a magnitude approaching that which distinguishes host species.

The contribution of the present study lies elsewhere. Because spatial subsampling was repeated across five surveys spanning nineteen months, we could ask whether the coherence of an individual plant as a microbial habitat is constant through time. It is not. Subsamples from the same plant were consistently more similar to one another than expected under random reassignment, confirming genuine plant-level structuring in every survey; yet the magnitude of this effect was substantially greater in the three high-richness surveys than in the two low-richness ones. The same contrast was recovered by a ratio-based control that cancels the mechanical dependence of Bray–Curtis dissimilarity on richness and persisted after excluding technically compromised samples. This refines existing sampling recommendations rather than overturning them: Laforest-Lapointe et al. [20] concluded that sampling a single consistent canopy position is sufficient when the objective is to quantify interspecific variation, a recommendation our high-richness surveys support, but our results counsel caution when communities are contracted, because it is precisely under those conditions that a single subsample is least representative of the individual from which it was taken.

The temporal pattern is also informative about the nature of the contraction itself. If the loss of taxa between surveys were deterministic, with all microsites converging on the same subset of tolerant lineages, within-plant coherence would be expected to increase as communities contract. The opposite was observed. The erosion of individual-level structure under contraction is instead consistent with stochastic loss, in which each microsite retains an idiosyncratic subset of the prior community. Deterministic host selection and stochastic contraction therefore appear to operate simultaneously in this system, with their relative weight shifting as the realised community expands and contracts.

The taxonomic composition recovered here is broadly consistent with the phyllosphere literature while showing features that may be characteristic of a high-altitude tropical setting, and the dominant lineages illustrate contrasting strategies for persisting on leaf surfaces. Beijerinckiaceae was the most abundant family (14.12%), driven by *Methylobacterium*–*Methylorubrum* (10.00%). These methylotrophs use the methanol released by pectin demethylation during plant cell wall metabolism, an association tightly coupled to leaf physiology and repeatedly identified as a defining feature of the phyllosphere habitat [7,26,27]. Their dominance indicates that the communities characterised are genuinely leaf-associated rather than dominated by transient atmospheric deposition. *Sphingomonas* (3.20%) represents a contrasting strategy within the same guild of pigmented Alphaproteobacteria: rather than exploiting a single abundant substrate, it deploys a broad repertoire of transporters that scavenge the scarce and heterogeneous carbon sources available on the leaf surface [26], and strains isolated from plants have been shown to suppress disease caused by foliar pathogens in gnotobiotic systems, an effect not observed for *Methylobacterium* [28]. The co-dominance of two lineages with such different resource strategies is consistent with a habitat in which distinct ecological niches coexist at small spatial scale.

*Pantoea* was the single most abundant genus (10.19%). Members of the Erwiniaceae and of the wider Enterobacterales are among the most extensively studied bacterial epiphytes, capable of rapid growth when leaf surface water and nutrients become transiently available and equipped with carotenoid pigmentation that contributes to persistence under solar radiation [6]. Their capacity for opportunistic proliferation bears directly on the temporal pattern we report: the low-richness samples of survey M4 were dominated by Enterobacterales at up to 96% relative abundance, which is the outcome expected when conditions favour a small number of rapidly dividing lineages at the expense of the remainder, and it illustrates how a nestedness-generating process can operate at the level of the individual leaf surface.

More distinctive is the combined abundance of *Deinococcus* (5.66%) and *Hymenobacter* (4.62%), which together accounted for 10.28% of the community. Both are carotenoid-pigmented lineages recognised for tolerance to ultraviolet radiation, oxidative stress and desiccation, and pigmentation has long been noted as a conspicuous feature of phyllosphere assemblages exposed to daily solar radiation [6]. Medellín lies at approximately 1,495 m above sea level, where ultraviolet irradiance is high throughout the year and lacks the seasonal modulation characteristic of temperate latitudes. A phyllosphere in which roughly one in ten sequences derives from radiation-tolerant lineages is consistent with sustained selection by ultraviolet exposure, and we propose this as a candidate distinguishing feature of tropical Andean urban phyllospheres. We emphasise that this inference rests on the taxonomic affiliation of the lineages recovered and not on any measurement of activity, pigmentation or ultraviolet dose; establishing a causal link would require functional or experimental evidence.

Host identity and urban zone together explained 31.8% of adjusted variation among plant-level community profiles, leaving 68.2% unexplained. The magnitude of within-plant heterogeneity documented here offers a partial account of this residual: because a substantial fraction of community variation occurs among microsites of a single individual on a single day, variation at that scale is necessarily unexplained by factors defined at the level of the plant, the zone or the survey. Combined with the finding that turnover dominates total dissimilarity, a high residual suggests a substantial contribution of stochastic assembly, including dispersal limitation, priority effects during colonisation and ecological drift within small local populations.

All of the associations reported here are observational. The design permits the contributions of host identity, urban space and time to be separated from one another, but not the mechanisms proposed to underlie them to be tested; these remain hypotheses that manipulative or trait-based studies would be required to evaluate.

Three limitations bound these conclusions. First, because the five surveys were separated by months, each corresponds to an independent round of DNA extraction, library preparation and sequencing, so the survey effect integrates ecological time and technical batch and the two cannot be separated statistically in this design, as discussed above. Second, the design comprises fifteen plants, five per host species and three per urban zone, which affords adequate power to detect the compositional effects reported but limited power for tests of multivariate dispersion and for detecting modest effects on alpha diversity, and precludes estimation of host species × urban zone interactions. Third, soil properties beneath the sampled plants were not characterised. Although the communities analysed here are strictly epiphytic and were recovered by surface washing of leaves processed on the day of collection, soil is a recognised source pool for phyllosphere colonists, and designs that pair leaf and soil sampling at the level of the individual plant would allow this contribution to be quantified.

## 4. Materials and Methods

### 4.1. Study Area and Sampling Design

The study was conducted in Medellín, Colombia (approximately 6°15′ N, 75°34′ W; 1495 m above sea level), a tropical Andean city located in the Aburrá Valley, a narrow valley of the Central Cordillera with a bimodal rainfall regime comprising wet seasons in April–May and September–November. Five urban zones were selected to represent the spatial heterogeneity of the city, each with a fixed sampling point located near a meteorological and air quality monitoring station: IDEA (Centro), Tránsito Caribe (Norte), Puente Aguacatala (Sur), Amsterdam Plaza (Este) and Biblioteca San Javier (Oeste).

The five zones were selected to span the cardinal sectors of the metropolitan area and for proximity to existing monitoring stations, rather than to maximise contrast in land use, traffic density or vegetation cover; in these respects, the five sampling points are broadly comparable. They differ consistently in microclimate, however: averaged across the five surveys, mean relative humidity ranged from 63.8% (Centro) to 78.1% (Este) and mean air temperature from 21.5 °C (Este) to 23.8 °C (Sur), with this ranking stable across every survey (Table S3). Mean air temperature and relative humidity for each sampling point on each survey day were obtained from the adjacent monitoring station of the Early Warning System of Medellín and the Aburrá Valley (SIATA). Quantitative air quality data recorded at these stations are analysed in a companion study and are not used here.

Within each urban zone, one individual plant of each of three widely planted species was selected and monitored throughout the study: *Mangifera indica* L. (mango; a tropical evergreen tree in the Anacardiaceae), *Terminalia catappa* L. (tropical almond; a tree in the Combretaceae) and *Thunbergia grandiflora* (Roxb. ex Rottler) Roxb. (blue trumpet vine; an evergreen vine in the Acanthaceae). The three species are widely cultivated ornamentals with distinctive morphology and had been identified in the field by members of the research project prior to the present study; no herbarium vouchers were deposited. The same individual plants were located and sampled in every survey at the five fixed sampling points listed above.

The study therefore comprised fifteen plants (3 host species × 5 urban zones), each sampled repeatedly in five surveys, hereafter referred to as surveys M1 to M5. Each survey consisted of a single collection day on which every plant was sampled: 21 September 2023 (M1), 1 December 2023 (M2), 11 April 2024 (M3), 28 November 2024 (M4) and 8 April 2025 (M5), spanning nineteen months (Table 1).

In every survey, six independent leaf samples of 50 g each were collected from different parts of the canopy of each plant, capturing within-individual spatial heterogeneity of the leaf surface. DNA was extracted from all six samples, and three were selected at random for sequencing; random selection was performed independently in each survey. The design therefore yields three spatial subsamples per plant and survey, giving 225 expected samples. One sample (04EA2; *T. catappa*, Este zone, survey M4) was not recovered after sequencing, so 224 samples were analysed.

This design defines a three-level hierarchy that determines the appropriate unit of inference for each factor. Host species and urban zone are between-plant factors, for which the experimental unit is the plant (n = 15); survey is a within-plant factor, for which the unit is the plant–survey combination (n = 75); and the three spatial subsamples characterise within-plant variation (n = 224). Statistical analyses were structured accordingly (Section 4.7). Because each urban zone contains a single plant of each host species, the identity of the individual plant is fully confounded with the host species × urban zone combination. Interactions between these two factors are therefore not estimable, and any effect attributed to host species or to urban zone incorporates whatever is idiosyncratic to the particular individual sampled.

### 4.2. Leaf Collection, Transportation and Recovery of Epiphytic Bacteria

Healthy leaves without visible damage, disease symptoms or pest presence were collected without petioles using gardening scissors disinfected with 70% ethanol between samples. Each field sample consisted of 50 g of leaves. Samples were placed in sterile resealable bags, transported to the laboratory under refrigeration (4–8 °C) and processed on the day of collection without freezing because freeze–thaw cycles rupture leaf tissue and release endophytic bacteria into the wash solution, which would compromise the specificity of the epiphytic fraction.

Epiphytic bacteria were recovered using a single standardised protocol applied identically across all surveys. Each 50 g leaf sample was placed in 100 mL of sterile 1× phosphate-buffered saline and bacteria were detached from the leaf surface by orbital shaking at 200 rpm for 10 min. The resulting suspension was recovered and centrifuged at 6000 rpm for 15 min in 50 mL conical tubes; when the suspension volume exceeded tube capacity, centrifugation was performed in two sequential rounds to concentrate the full volume into a single pellet.

Total DNA was extracted from the bacterial pellet using the QIAamp PowerFecal Pro kit (QIAGEN, Hilden, Germany) following the manufacturer’s instructions. DNA concentration and purity were assessed with a NanoDrop OneC spectrophotometer (Thermo Fisher Scientific, Waltham, MA, USA), using the A260/A280 and A260/A230 absorbance ratios as purity indicators. No extraction blanks or no template amplification controls were processed; the implications are addressed in Section 3 and evaluated through the sensitivity analysis described in Section 4.8.

### 4.3. 16S rRNA Gene Amplicon Sequencing

Library preparation and sequencing were performed by Macrogen Inc. (Seoul, Republic of Korea). The V3–V4 hypervariable region of the bacterial 16S rRNA gene was amplified using the primers 341F (5′-CCTACGGGNGGCWGCAG-3′) and 805R (5′-GACTACHVGGGTATCTAATCC-3′). Libraries were prepared according to the provider’s standard protocol and sequenced on an Illumina MiSeq platform (Illumina, San Diego, CA, USA) using 300 bp paired-end reads, with a target output of approximately 500,000 read pairs per sample. Samples from the five surveys were submitted in separate batches as each survey was completed; the survey factor is therefore confounded with extraction, library preparation and sequencing batch.

### 4.4. Sequence Processing and ASV Inference

Raw paired-end reads were processed in QIIME 2 v2025.2 [29] and imported using a PairedEndFastqManifestPhred33V2 manifest. Primer sequences were removed with the q2-cutadapt plugin (cutadapt trim-paired). Trimmed reads were denoised with DADA2 [30] as implemented in q2-dada2 (denoise-paired). Left trimming was set to zero because primers had already been removed by cutadapt. This workflow yielded 168,120 ASVs across 224 samples.

### 4.5. Taxonomic Assignment and Phylogenetic Reconstruction

Taxonomy was assigned with a naïve Bayes classifier [31] trained on the SILVA 138 SSURef_NR99 reference database [32]. The reference was retrieved and curated with RESCRIPt [33]: sequences and taxonomy were obtained with get-silva-data (version 138, target SSURef_NR99, species labels included), culled of low-quality sequences, filtered by taxon-specific length thresholds (filter-seqs-length-by-taxon; minimum lengths of 900, 1200 and 1400 nucleotides for Archaea, Bacteria and Eukaryota, respectively) and dereplicated in uniq mode using SILVA rank handles. The V3–V4 region was extracted with extract-reads using the same primer pair (--p-identity 0.8, --p-min-length 50, forward read orientation), and the classifier was fitted with fit-classifier-naive-bayes. Classification was performed with classify-sklearn at a confidence threshold of 0.7. A rooted phylogenetic tree was constructed with the align-to-tree-mafft-fasttree pipeline (MAFFT alignment, positional masking, FastTree inference, midpoint rooting). Chloroplast and mitochondrial sequences were removed prior to tree construction, yielding a tree with 161,758 tips.

### 4.6. Dataset Construction and Filtering

Feature table, taxonomy, rooted tree and sample metadata were imported into R v4.5.1 with qiime2R v0.99.6 and combined into a phyloseq object [34]. Retaining only ASVs assigned to the domain Bacteria excluded Archaea (39), Eukaryota (1) and features unassigned at domain level (53), yielding 161,665 bacterial ASVs and 44,325,146 reads. Because 130,244 bacterial ASVs (80.6%) were detected in a single sample yet accounted for only 2.52% of reads, a distribution consistent with residual denoising noise rather than with biologically meaningful rare taxa, an ecological filter retaining ASVs present in at least two samples and represented by at least ten reads was applied, yielding 29,894 ASVs and 43,197,788 reads (97.46% of bacterial reads). All analyses use this single filtered dataset, so that alpha and beta diversity are estimated from identical underlying data.

Alpha diversity was computed after rarefaction to 45,000 reads per sample without replacement. This depth was chosen because it retained all 224 samples: the minimum sequencing depth in the filtered table was 49,216 reads, and rarefaction to 50,000 would have excluded one sample. Rarefaction curves confirmed that richness approached saturation well below this depth in all samples (Figure S1). A single random seed (123) was used throughout the analysis.

Relative abundances for taxonomic description were calculated within each sample and then averaged across samples within each group, so that every sample contributes equally regardless of its sequencing depth. Taxa were summarised at phylum, family and genus level; for visualisation, the most abundant taxa across the entire dataset were retained and the remainder pooled as “Other”, using a single global selection so that colour assignments remain comparable across panels.

### 4.7. Statistical Analysis and Levels of Inference

To avoid pseudoreplication, each factor was tested at the level at which it varies.

Alpha diversity. Observed ASV richness, Chao1, Shannon, Simpson and inverse Simpson indices were calculated with phyloseq, and Faith’s phylogenetic diversity with picante [35]. Metrics were averaged across the three spatial subsamples within each plant–survey combination (n = 75) and modelled with a linear mixed-effects model in lme4 [36] including host species, urban zone and survey as fixed effects and a random intercept for plant identity. Type III *F*-tests with Satterthwaite approximation of degrees of freedom were obtained with lmerTest [37]; the variance of the random intercept, the intraclass correlation coefficient and whether the fit was singular are reported for each model. Because host species and urban zone are between-plant factors, they were additionally tested on plant-level means (n = 15) by Type II analysis of variance. Post hoc pairwise contrasts among surveys were obtained with emmeans and adjusted using the Benjamini–Hochberg procedure. Marginal effect sizes are reported as η^2^. Associations between survey-level meteorological variables and diversity metrics were assessed by Spearman rank correlation across the five surveys.

Beta diversity. Community dissimilarity was quantified with five complementary metrics. Bray–Curtis and binary Jaccard distances were computed on relative abundances; Aitchison distances as Euclidean distances on centred log-ratio transformed counts with a pseudocount of one [38]; weighted and unweighted UniFrac distances on relative abundances using the rooted phylogeny. Conversion to relative abundance before distance calculation was applied because sequencing depth spanned a 21-fold range. For host species and urban zone, community profiles were aggregated to plant level by summing counts across all subsamples and surveys and rarefying to the minimum plant-level depth; marginal PERMANOVA [39] was then applied to the fifteen plant profiles with 9999 permutations. For survey, all 224 samples were retained and permutations were restricted within plants using the permute package, respecting the repeated-measures structure. Homogeneity of multivariate dispersion was tested with betadisper followed by permutation tests at the corresponding level. Principal coordinates analysis of Bray–Curtis dissimilarities was used for ordination; the ordination is presented for visualisation only, and all tests of group separation operate on the full distance matrix rather than on a reduced-dimensional projection.

Variance partitioning was performed with varpart on plant-level Hellinger-transformed profiles [40] with host species and urban zone as explanatory blocks; unique fractions were estimated by partial redundancy analysis and are reported as adjusted *R*^2^.

Beta diversity decomposition. Jaccard dissimilarity was partitioned into turnover and nestedness components following Baselga [14] using beta.pair in the betapart package [41]. For host species and urban zone the decomposition was computed on plant-level rarefied profiles; for survey it was computed on sample-level rarefied profiles with permutations restricted within plants. Because the nestedness component of Jaccard dissimilarity is not a metric distance, permutational tests on this matrix can return negative sums of squares and correspondingly uninterpretable *R*^2^ and *F* values; such results are reported as absence of effect, and the comparison of component means, which is not subject to this limitation, is treated as the primary evidence.

Temporal decay of community similarity was evaluated by regressing Bray–Curtis dissimilarity against days elapsed between samplings, restricted to pairs of subsamples from the same plant, and quantified with Spearman correlation.

### 4.8. Within-Plant Heterogeneity and Technical Assessment

Bray–Curtis dissimilarities were classified into four hierarchical scales: between subsamples of the same plant in the same survey; between subsamples of the same plant in different surveys; between plants of the same host species in different zones within a survey; and between plants of different host species in the same zone within a survey.

Because Bray–Curtis dissimilarity increases mechanically as richness declines, three independent controls were applied to the relationship between within-plant heterogeneity and community richness. First, the ratio of within-plant to between-plant dissimilarity within each survey, which cancels the shared mechanical inflation. Second, recomputation excluding the twelve low-richness samples of survey M4. Third, a null model in which subsamples were randomly reassigned to plants within each survey (999 randomisations), preserving each sample’s observed richness; the standardised effect size quantifies how much more similar subsamples of the same plant are than expected by chance.

Because survey is confounded with technical batch, sequencing depth per survey, the number of survey-exclusive ASVs, the relative abundance of the most abundant ASV per sample and the number of samples yielding fewer than 100 observed ASVs were quantified. The correlation between sequencing depth and ordination axes was computed before and after rarefaction; an unchanged correlation indicates that any association reflects confounding with survey rather than a depth artefact. All analyses were repeated excluding the twelve low-richness samples, and, in the absence of extraction blanks, the relative abundance of genera frequently reported as reagent contaminants [42] was quantified.

### 4.9. Software and Reproducibility

Analyses were performed in R v4.5.1 using phyloseq v1.52.0, vegan v2.7.1, lme4, lmerTest, emmeans, picante v1.8.2, betapart v1.6.1, permute and qiime2R v0.99.6. A single random seed (123) was used throughout. Figures were produced with ggplot2 and patchwork.

## 5. Conclusions

Epiphytic phyllosphere bacterial communities of urban plants in a tropical Andean city were structured by host plant identity, urban spatial heterogeneity and sampling survey, and these filters appear to operate through distinct mechanisms. Host identity and urban zone act through compositional turnover, replacing taxa without altering the amount of diversity a leaf supports; survey acts through nestedness, driving a fivefold contraction of richness without substantially changing community identity, a contraction that tracked a monotonic rise in relative humidity across the study period. The two spatial filters explained statistically independent fractions of variation, and host identity accounted for the larger unique fraction once model complexity was taken into account.

A fourth scale of variation proved quantitatively important: bacterial communities from different parts of a single canopy differed by 69% of the dissimilarity separating different host species. Individual plants were nevertheless detectably coherent as microbial habitats, with subsamples more similar than expected by chance in every survey, although this coherence eroded as communities contracted—a pattern consistent with stochastic rather than deterministic loss of taxa.

Four constraints bound these conclusions. Each survey corresponds to an independent round of DNA extraction, library preparation and sequencing, so the survey effect integrates ecological time and technical batch and is best regarded as an upper bound on temporal ecological variation; the association with relative humidity is correspondingly correlational rather than causal. No extraction blanks were processed, so reagent-derived contamination cannot be formally excluded, although the genera most frequently reported as kit contaminants were essentially absent. Because a single plant of each species was monitored in each zone, individual plant identity is confounded with the species × zone combination, so the effects reported are not formally separable from the idiosyncrasy of the fifteen individuals sampled. Finally, soil properties beneath the sampled plants were not characterised, and designs pairing leaf and soil sampling at the level of the individual plant would allow for the contribution of this source pool to be quantified.

Together, these results suggest that deterministic host selection and stochastic temporal contraction may shape urban phyllosphere assembly through complementary rather than redundant pathways, and they provide a baseline for tropical Andean urban systems whose seasonal and radiative regimes differ substantially from those of the temperate ecosystems that have shaped the expectations of the field.

## Figures and Tables

**Table 1:** Sampling design and meteorological conditions. Each survey consisted of a single collection day on which all fifteen plants were sampled. Air temperature and relative humidity are means across the five sampling points, each of which is adjacent to a monitoring station of the Early Warning System of Medellín and the Aburrá Valley (SIATA); zone-level values are given in Table S3. Three spatial subsamples were sequenced per plant and survey; of the 225 samples expected, 224 were recovered.

Survey	Date	Mean Air Temperature (°C)	Mean Relative Humidity (%)	Samples
M1	21 September 2023	23.2	58.0	45
M2	1 December 2023	22.9	63.1	45
M3	11 April 2024	23.5	66.9	45
M4	28 November 2024	22.3	71.6	44
M5	8 April 2025	21.6	77.9	45

**Table 2:** Community dissimilarity across the nested scales of the sampling design. Values are medians and means of Bray–Curtis dissimilarity for all pairwise comparisons within each scale, together with the number of pairs. The lower section reports, for each sample, the median distance to three reference sets, and the paired comparison between the host species contrast and the spatial contrast (Wilcoxon signed-rank test). Aitchison distances gave equivalent results.

Analysis	Contrast	n	Median Bray–Curtis Dissimilarity	Mean Bray–Curtis Dissimilarity
Hierarchical pairwise comparison	Same plant, same survey	223	0.542	0.584
Hierarchical pairwise comparison	Same plant, different survey	1338	0.760	0.756
Hierarchical pairwise comparison	Same host species, different urban zone, same survey	1338	0.761	0.770
Hierarchical pairwise comparison	Different host species, same urban zone, same survey	669	0.782	0.773
Hierarchical pairwise comparison	Remaining pairwise comparisons	21,408	0.854	0.841
Matched contrast	Same plant, same survey	224	0.548	0.585
Matched contrast	Same host species, different urban zone	224	0.755	0.776
Matched contrast	Different host species, same urban zone	224	0.772	0.782

**Table 3:** Linear mixed-effects models of alpha diversity fitted at the plant–survey level (n = 75), with host species, urban zone and survey as fixed effects and a random intercept for plant identity. For each metric the table gives *F* statistics, numerator and denominator degrees of freedom (Satterthwaite approximation), *p*-values, the intraclass correlation coefficient for plant identity and whether the model fit was singular. Denominator degrees of freedom for host species and urban zone are 8, as expected for between-plant factors in a design of fifteen plants. Results of the complementary plant-level analysis (n = 15) are given in Table S7.

Alpha Diversity Metric	Fixed Effect	F	df	*p*-Value	ICC	Singular Fit
Observed ASVs	Host plant species	0.18	2, 8	0.837	0.058	No
	Urban zone	0.90	4, 8	0.508	0.058	No
	Survey	63.73	4, 56	<0.001	0.058	No
Chao1 richness	Host plant species	0.18	2, 8	0.838	0.062	No
	Urban zone	0.76	4, 8	0.579	0.062	No
	Survey	69.19	4, 56	<0.001	0.062	No
Shannon diversity	Host plant species	0.52	2, 64	0.598	0.000	Yes
	Urban zone	2.45	4, 64	0.055	0.000	Yes
	Survey	30.87	4, 64	<0.001	0.000	Yes
Simpson diversity	Host plant species	0.30	2, 64	0.744	0.000	Yes
	Urban zone	2.88	4, 64	0.029	0.000	Yes
	Survey	13.01	4, 64	<0.001	0.000	Yes
Inverse Simpson diversity	Host plant species	1.57	2, 64	0.217	0.000	Yes
	Urban zone	0.41	4, 64	0.800	0.000	Yes
	Survey	9.62	4, 64	<0.001	0.000	Yes
Faith’s phylogenetic diversity	Host plant species	0.40	2, 8	0.681	0.025	No
	Urban zone	3.06	4, 8	0.083	0.025	No
	Survey	63.09	4, 56	<0.001	0.025	No

**Table 4:** Marginal PERMANOVA of community composition across five distance metrics, with 9999 permutations. Host species and urban zone were tested on plant-level community profiles (n = 15) obtained by summing counts within each plant and rarefying to the minimum plant-level depth; survey was tested on the 224 individual samples with permutations restricted within plants. *R*^2^ values are comparable among metrics within a factor but not in absolute magnitude between the two levels of analysis.

Beta-Diversity Metric	Factor	Analytical Level	R^2^	F	*p*-Value
Bray–Curtis	Host plant species	Plant-level profiles (n = 15)	0.301	3.37	<0.001
Bray–Curtis	Urban zone	Plant-level profiles (n = 15)	0.343	1.93	<0.001
Jaccard	Host plant species	Plant-level profiles (n = 15)	0.193	1.73	<0.001
Jaccard	Urban zone	Plant-level profiles (n = 15)	0.361	1.62	<0.001
Aitchison/CLR	Host plant species	Plant-level profiles (n = 15)	0.222	2.15	<0.001
Aitchison/CLR	Urban zone	Plant-level profiles (n = 15)	0.363	1.75	<0.001
Weighted UniFrac	Host plant species	Plant-level profiles (n = 15)	0.253	2.62	<0.001
Weighted UniFrac	Urban zone	Plant-level profiles (n = 15)	0.360	1.86	0.002
Unweighted UniFrac	Host plant species	Plant-level profiles (n = 15)	0.203	1.89	0.002
Unweighted UniFrac	Urban zone	Plant-level profiles (n = 15)	0.368	1.71	<0.001
Bray–Curtis	Survey	Sample-level profiles; permutations restricted within plant	0.098	5.97	<0.001
Jaccard	Survey	Sample-level profiles; permutations restricted within plant	0.096	5.79	<0.001
Aitchison/CLR	Survey	Sample-level profiles; permutations restricted within plant	0.119	7.41	<0.001
Weighted UniFrac	Survey	Sample-level profiles; permutations restricted within plant	0.053	3.05	<0.001
Unweighted UniFrac	Survey	Sample-level profiles; permutations restricted within plant	0.098	5.92	<0.001

**Table 5:** Variance partitioning of Hellinger-transformed plant-level community profiles (n = 15) between host species and urban zone. Unique fractions were estimated by partial redundancy analysis with the remaining factor as a conditioning variable and are expressed as adjusted *R*^2^. Negative values of the shared fraction are an arithmetic property of adjusted *R*^2^ and are interpreted as absence of shared variation.

Variance Fraction	Adjusted R^2^	Variation (%)
Host species (unique)	0.236	23.6
Urban zone (unique)	0.174	17.4
Shared	−0.092	−9.2
Total explained	0.318	31.8
Residual	0.682	68.2

**Table 6:** Partitioning of Jaccard dissimilarity into turnover and nestedness components. The upper section gives marginal PERMANOVA results for each component analysed separately; the lower section gives mean component values within and between groups for each factor. Host species and urban zone were analysed on plant-level rarefied profiles (n = 15) and survey on individual samples with permutations restricted within plants. Because the nestedness component of Jaccard dissimilarity is not a metric distance, permutational tests on this matrix can return negative sums of squares and correspondingly uninterpretable *R*^2^ and *F* values; these are reported as absence of effect and the component means constitute the primary evidence.

Factor	Component	Analytical Level	R^2^	F	*p*-Value	Mean Within Groups	Mean Between Groups
Host species	Turnover	Plant level (*n* = 15)	0.208	2.02	<0.001	0.663	0.699
Host species	Nestedness	Plant level (*n* = 15)	−0.099	−0.38	0.916	0.043	0.035
Host species	Total Jaccard	Plant level (*n* = 15)	0.193	1.73	<0.001	—	—
Urban zone	Turnover	Plant level (*n* = 15)	0.379	1.83	<0.001	0.642	0.697
Urban zone	Nestedness	Plant level (*n* = 15)	0.052	0.10	0.869	0.044	0.036
Urban zone	Total Jaccard	Plant level (*n* = 15)	0.361	1.62	<0.001	—	—
Survey	Turnover	Sample level; permutations restricted within plant	−0.008	−0.44	1.000	—	—
Survey	Nestedness	Sample level; permutations restricted within plant	0.508	56.61	<0.001	—	—

## Data Availability

Raw demultiplexed sequence data have been deposited in the NCBI Sequence Read Archive under BioProject accession PRJNA1531952.

## Supplementary Materials

The following supporting information can be downloaded at https://www.mdpi.com/article/10.3390/plants15203202/s1, Figure S1: Rarefaction curves; Figure S2: Technical assessment of the sampling survey factor; Figure S3: Family- and genus-level composition by host species; Figure S4: Family- and genus-level composition by survey; Figure S5: Community distance contrasts under matched conditions; Figure S6: Comparison of alpha diversity on filtered and unfiltered datasets; Figure S7: Alpha diversity by host species and urban zone; Figure S8: Principal coordinates analysis including the third coordinate; Figure S9: Multivariate dispersion; Figure S10: Temporal decay of community similarity; Table S1: Non-target ASV filtering and taxonomic classification completeness of the bacterial dataset; Table S2: Sequencing depth retained reads and filtered ASVs per sample; Table S3: Meteorological conditions by urban zone and survey; Table S4: Technical diagnostics by survey; Table S5: Relationship between sequencing depth and Bray–Curtis ordination axes before and after rarefaction; Table S6: Global taxonomic composition of epiphytic phyllosphere bacterial communities and survey-specific abundance of Enterobacteriaceae; Table S7: Plant-level alpha-diversity tests for host species and urban zone (n = 15); Table S8: Post hoc pairwise contrasts among surveys for alpha-diversity metrics; Table S9: Tests for homogeneity of multivariate dispersion across host species, urban zones, and surveys; Table S10: Bray–Curtis dissimilarity across temporal intervals between surveys; Table S11: Within-plant heterogeneity and null-model controls of plant-level community coherence; Table S12: Sensitivity analyses and screening of potential contaminant-associated genera.

## References

[1] Lu C., Xiao Z., Li H., Han R., Sun A., Xiang Q., Zhu Z., Li G., Yang X., Zhu Y.-G. (2024). Aboveground plants determine the exchange of pathogens within air-phyllosphere-soil continuum in urban greenspaces. J. Hazard. Mater..

[2] Mhuireach G., Betancourt-Román C.M., Green J.L., Johnson B.R. (2019). Spatiotemporal controls on the urban aerobiome. Front. Ecol. Evol..

[3] Sohrabi R., Paasch B.C., Liber J.A., He S.Y. (2023). Phyllosphere microbiome. Annu. Rev. Plant Biol..

[4] Zhang Y., Li X., Lu L., Huang F., Liu H., Zhang Y., Yang L., Usman M., Li S. (2023). Urbanization reduces phyllosphere microbial network complexity and species richness of camphor trees. Microorganisms.

[5] Espenshade J., Thijs S., Gawronski S., Bové H., Weyens N., Vangronsveld J. (2019). Influence of urbanization on epiphytic bacterial communities of the *Platanus* × *hispanica* tree leaves in a biennial study. Front. Microbiol..

[6] Lindow S.E., Brandl M.T. (2003). Microbiology of the phyllosphere. Appl. Environ. Microbiol..

[7] Vorholt J.A. (2012). Microbial life in the phyllosphere. Nat. Rev. Microbiol..

[8] Bulgarelli D., Schlaeppi K., Spaepen S., van Themaat E.V.L., Schulze-Lefert P. (2013). Structure and functions of the bacterial microbiota of plants. Annu. Rev. Plant Biol..

[9] Zhai L., Yang J., Lu M., Sun T., Wang Y., Tang G., Wu D., Xu L. (2025). Effects of leaf structure, physiological characteristics, and chemical properties on phyllosphere microorganisms associated with four forage crops in fallow land. Microb. Ecol..

[10] Mechan-Llontop M.E., Mullet J., Shade A. (2023). Phyllosphere exudates select for distinct microbiome members in sorghum epicuticular wax and aerial root mucilage. Phytobiomes J..

[11] Laforest-Lapointe I., Messier C., Kembel S.W. (2016). Host species identity, site and time drive temperate tree phyllosphere bacterial community structure. Microbiome.

[12] Redford A.J., Bowers R.M., Knight R., Linhart Y., Fierer N. (2010). The ecology of the phyllosphere: Geographic and phylogenetic variability in the distribution of bacteria on tree leaves. Environ. Microbiol..

[13] Kembel S.W., O’Connor T.K., Arnold H.K., Hubbell S.P., Wright S.J., Green J.L. (2014). Relationships between phyllosphere bacterial communities and plant functional traits in a neotropical forest. Proc. Natl. Acad. Sci. USA.

[14] Baselga A. (2010). Partitioning the turnover and nestedness components of beta diversity. Glob. Ecol. Biogeogr..

[15] Yan Z.Z., Chen Q.L., Li C.Y., Thi Nguyen B.A., He J.Z., Hu H.W. (2022). Contrasting ecological processes shape the *Eucalyptus* phyllosphere bacterial and fungal community assemblies. J. Sustain. Agric. Environ..

[16] Copeland J.K., Yuan L., Layeghifard M., Wang P.W., Guttman D.S. (2015). Seasonal community succession of the phyllosphere microbiome. Mol. Plant-Microbe Interact..

[17] Maignien L., DeForce E.A., Chafee M.E., Eren A.M., Simmons S.L. (2014). Ecological succession and stochastic variation in the assembly of *Arabidopsis thaliana* phyllosphere communities. mBio.

[18] Stone B.W.G., Jackson C.R. (2020). Seasonal patterns contribute more towards phyllosphere bacterial community structure than short-term perturbations. Microb. Ecol..

[19] Leff J.W., del Tredici P., Friedman W.E., Fierer N. (2015). Spatial structuring of bacterial communities within individual *Ginkgo biloba* trees. Environ. Microbiol..

[20] Laforest-Lapointe I., Messier C., Kembel S.W. (2016). Tree phyllosphere bacterial communities: Exploring the magnitude of intra- and inter-individual variation among host species. PeerJ.

[21] Boutin S., Laforest-Lapointe I. (2025). Reproducing plant microbiome research reveals site and time as key drivers of apple tree phyllosphere bacterial communities. Sci. Rep..

[22] Meyer K.M., Porch R., Muscettola I.E., Vasconcelos A.L.S., Sherman J.K., Metcalf C.J.E., Lindow S.E., Koskella B. (2022). Plant neighborhood shapes diversity and reduces interspecific variation of the phyllosphere microbiome. ISME J..

[23] Wuyts K., Smets W., Lebeer S., Samson R. (2020). Green infrastructure and atmospheric pollution shape diversity and composition of phyllosphere bacterial communities in an urban landscape. FEMS Microbiol. Ecol..

[24] Perreault R., Laforest-Lapointe I. (2022). Plant-microbe interactions in the phyllosphere: Facing challenges of the Anthropocene. ISME J..

[25] Antunes L.P., Martins L.F., Pereira R.V., Thomas A.M., Barbosa D., Lemos L.N., Silva G.M.M., Moura L.M.S., Epamino G.W.C., Digiampietri L.A. (2016). Microbial community structure and dynamics in thermophilic composting viewed through metagenomics and metatranscriptomics. Sci. Rep..

[26] Delmotte N., Knief C., Chaffron S., Innerebner G., Roschitzki B., Schlapbach R., von Mering C., Vorholt J.A. (2009). Community proteogenomics reveals insights into the physiology of phyllosphere bacteria. Proc. Natl. Acad. Sci. USA.

[27] Knief C., Delmotte N., Chaffron S., Stark M., Innerebner G., Wassmann R., von Mering C., Vorholt J.A. (2012). Metaproteogenomic analysis of microbial communities in the phyllosphere and rhizosphere of rice. ISME J..

[28] Innerebner G., Knief C., Vorholt J.A. (2011). Protection of *Arabidopsis thaliana* against leaf-pathogenic *Pseudomonas syringae* by *Sphingomonas* strains in a controlled model system. Appl. Environ. Microbiol..

[29] Bolyen E., Rideout J.R., Dillon M.R., Bokulich N.A., Abnet C.C., Al-Ghalith G.A., Alexander H., Alm E.J., Arumugam M., Asnicar F. (2019). Reproducible, interactive, scalable and extensible microbiome data science using QIIME 2. Nat. Biotechnol..

[30] Callahan B.J., McMurdie P.J., Rosen M.J., Han A.W., Johnson A.J.A., Holmes S.P. (2016). DADA2: High-resolution sample inference from Illumina amplicon data. Nat. Methods.

[31] Bokulich N.A., Kaehler B.D., Rideout J.R., Dillon M., Bolyen E., Knight R., Huttley G.A., Caporaso J.G. (2018). Optimizing taxonomic classification of marker-gene amplicon sequences with QIIME 2’s q2-feature-classifier plugin. Microbiome.

[32] Quast C., Pruesse E., Yilmaz P., Gerken J., Schweer T., Yarza P., Peplies J., Glöckner F.O. (2013). The SILVA ribosomal RNA gene database project: Improved data processing and web-based tools. Nucleic Acids Res..

[33] Robeson M.S., O’Rourke D.R., Kaehler B.D., Ziemski M., Dillon M.R., Foster J.T., Bokulich N.A. (2021). RESCRIPt: Reproducible sequence taxonomy reference database management. PLoS Comput. Biol..

[34] McMurdie P.J., Holmes S. (2013). phyloseq: An R package for reproducible interactive analysis and graphics of microbiome census data. PLoS ONE.

[35] Kembel S.W., Cowan P.D., Helmus M.R., Cornwell W.K., Morlon H., Ackerly D.D., Blomberg S.P., Webb C.O. (2010). picante: R tools for integrating phylogenies and ecology. Bioinformatics.

[36] Bates D., Mächler M., Bolker B.M., Walker S.C. (2015). Fitting linear mixed-effects models using lme4. J. Stat. Softw..

[37] Kuznetsova A., Brockhoff P.B., Christensen R.H.B. (2017). lmerTest package: Tests in linear mixed effects models. J. Stat. Softw..

[38] Gloor G.B., Macklaim J.M., Pawlowsky-Glahn V., Egozcue J.J. (2017). Microbiome datasets are compositional: And this is not optional. Front. Microbiol..

[39] Anderson M.J. (2001). A new method for non-parametric multivariate analysis of variance. Austral Ecol..

[40] Legendre P., Gallagher E.D. (2001). Ecologically meaningful transformations for ordination of species data. Oecologia.

[41] Baselga A., Orme C.D.L. (2012). betapart: An R package for the study of beta diversity. Methods Ecol. Evol..

[42] Salter S.J., Cox M.J., Turek E.M., Calus S.T., Cookson W.O., Moffatt M.F., Turner P., Parkhill J., Loman N.J., Walker A.W. (2014). Reagent and laboratory contamination can critically impact sequence-based microbiome analyses. BMC Biol..

