# Supplementary material for: Host Identity and Urban Space Structure Phyllosphere Bacterial Communities Through Compositional Turnover in a Tropical Andean City
Source: Plants (Basel). 2026 Oct 19;15(20):3202. doi: 10.3390/plants15203202 (PMC13708965; doi:10.3390/plants15203202)
Supplement: Supplementary file 1 [file plants-15-03202-s001.zip › plants-4506568-Supplementary Figures.pdf]

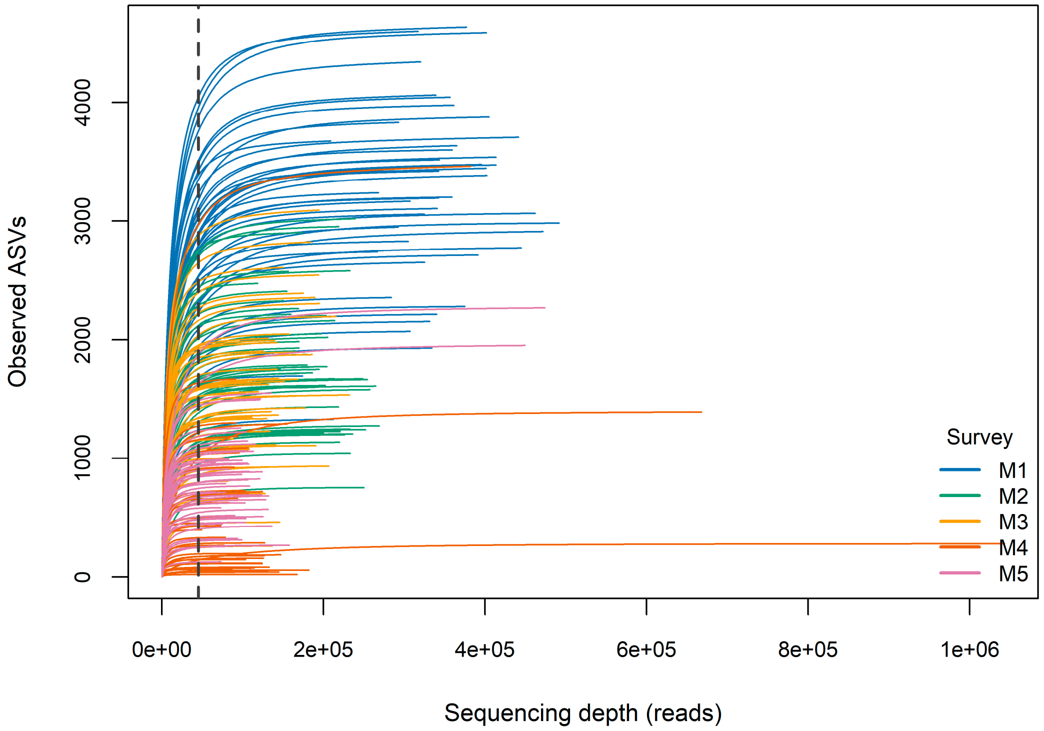

Figure S1. Rarefaction curves.

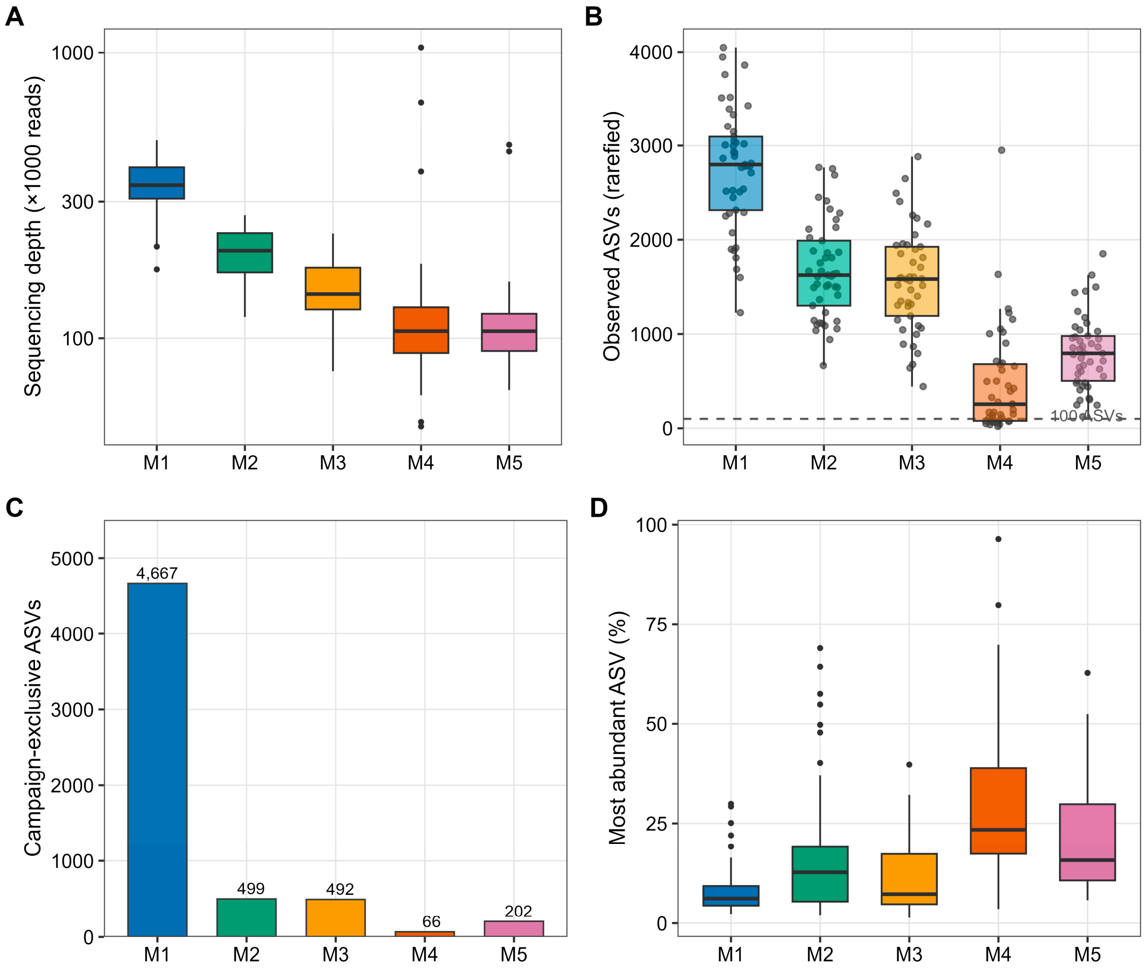

Figure S2. Technical assessment of the sampling survey factor.

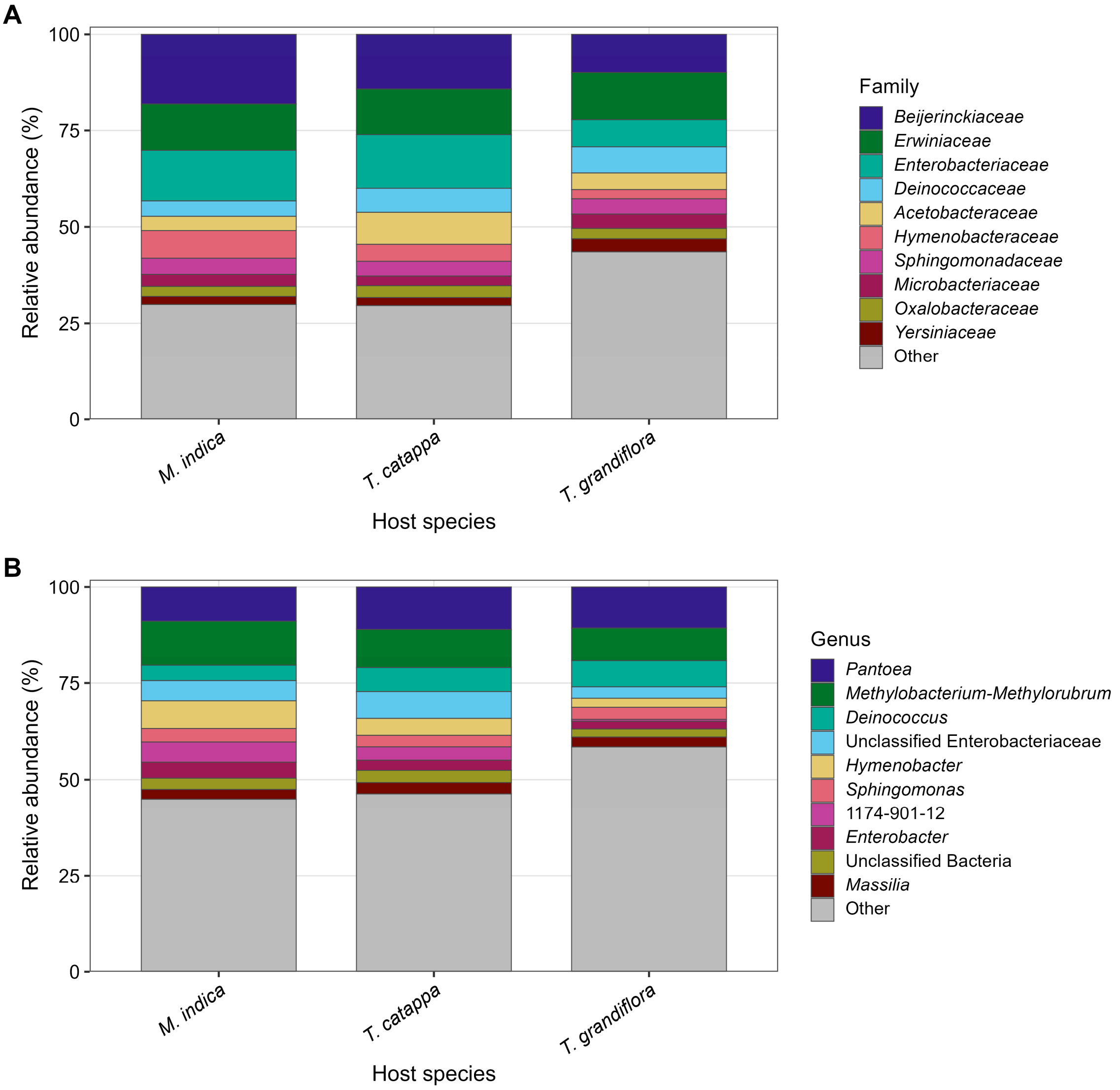

Figure S3. Family- and genus-level composition by host species.

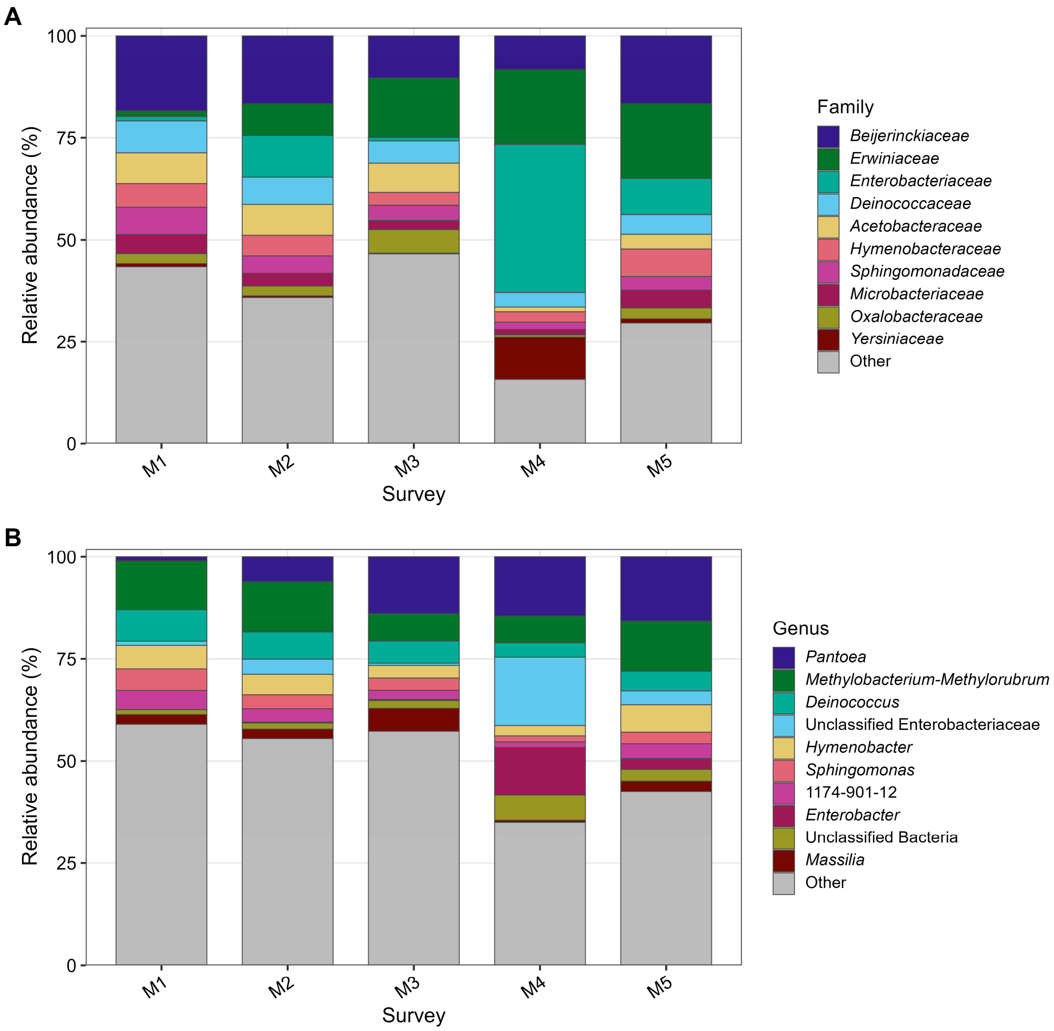

Figure S4. Family- and genus-level composition by survey.

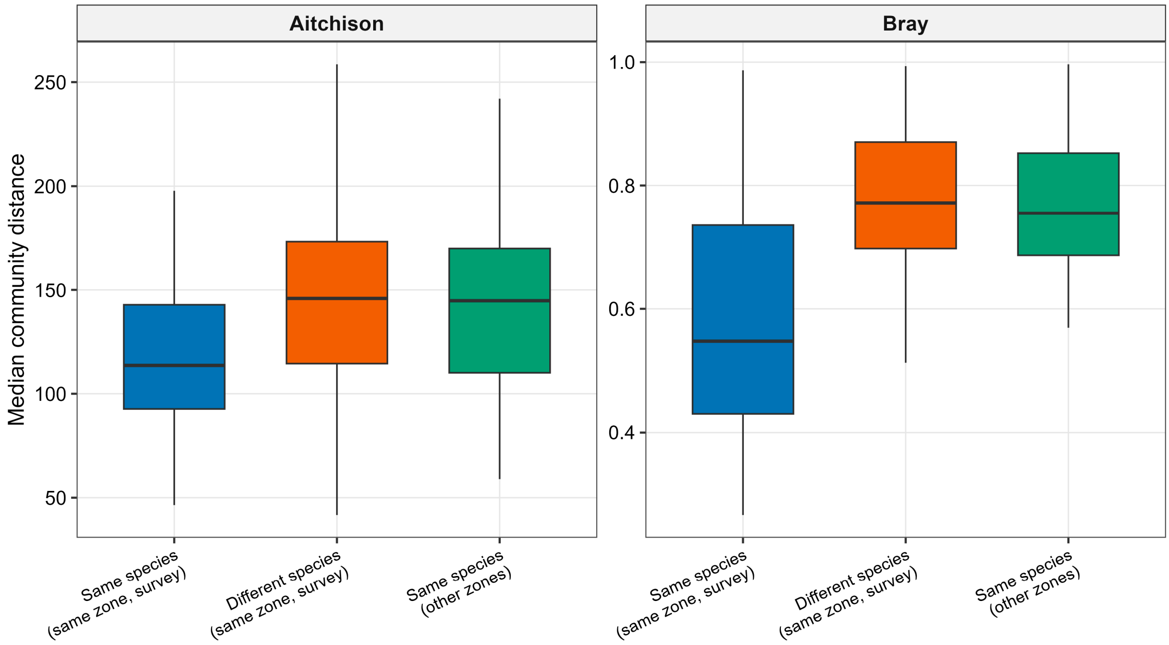

Figure S5. Community distance contrasts under matched conditions.

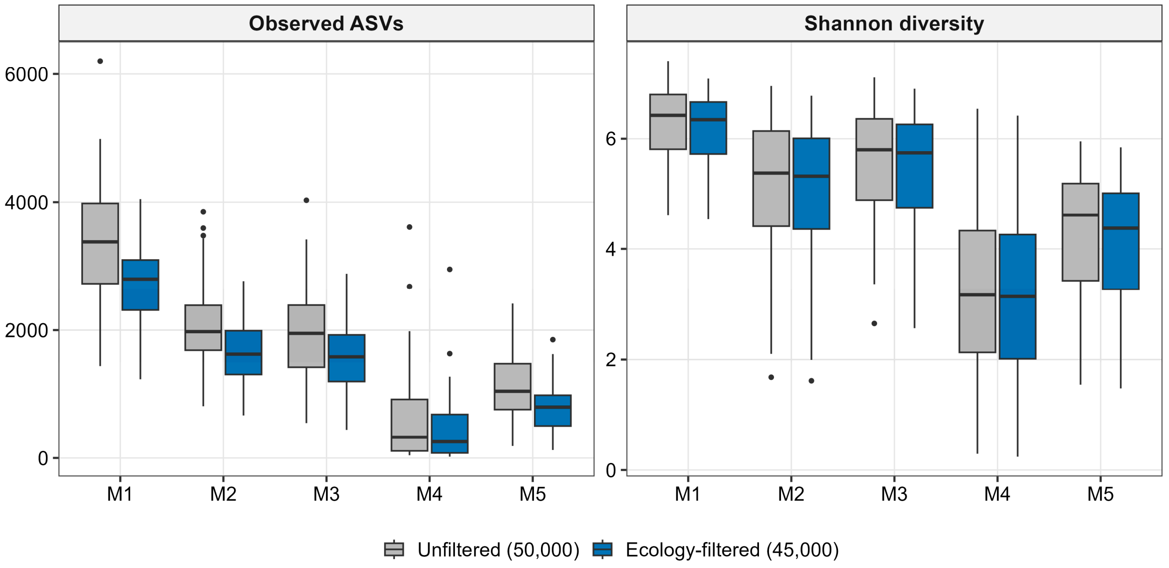

Figure S6. Comparison of alpha diversity on filtered and unfiltered datasets.

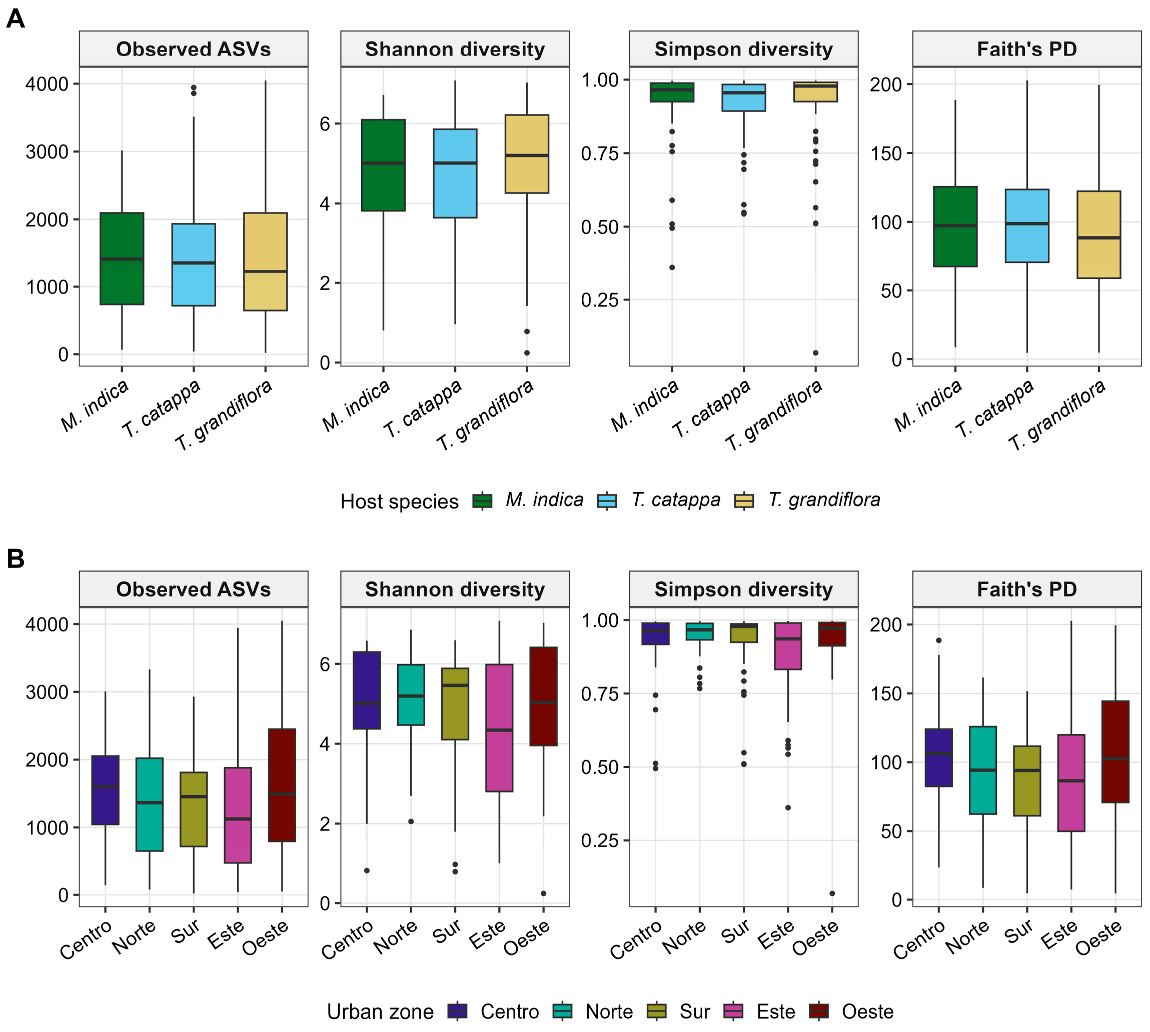

Figure S7. Alpha diversity by host species and urban zone.

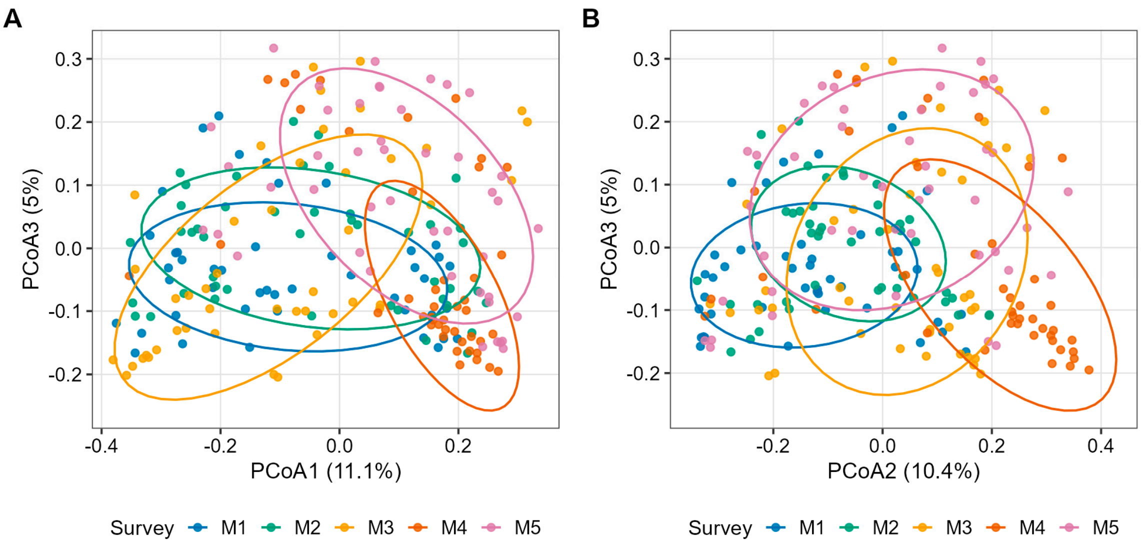

Figure S8. Principal coordinates analysis including the third coordinate.

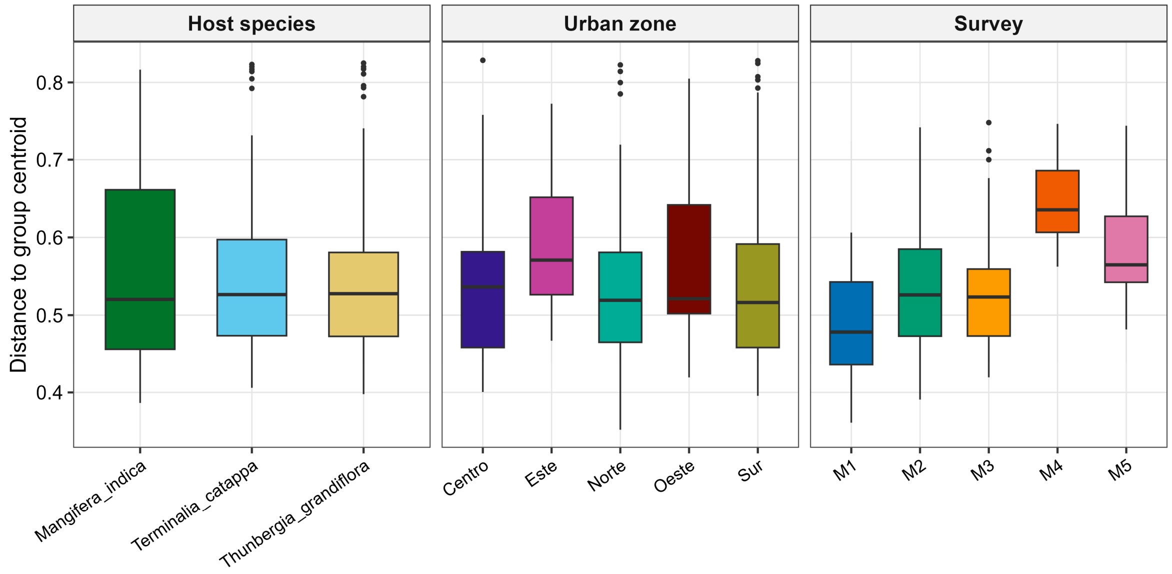

Figure S9. Multivariate dispersion;.

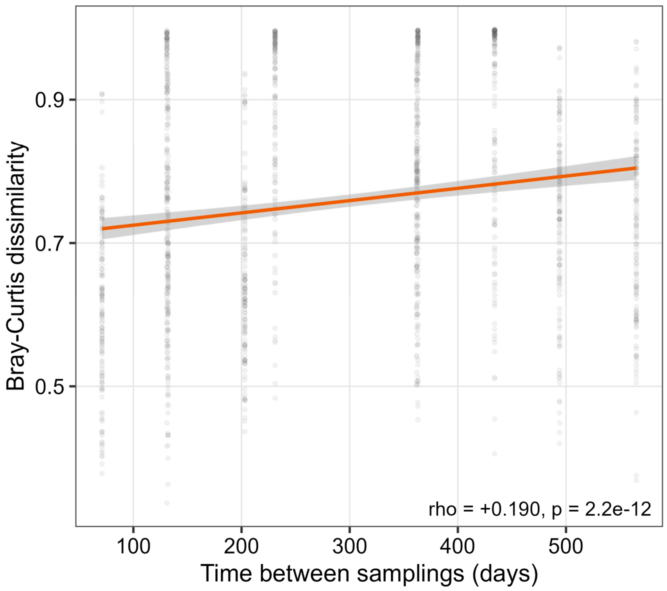

Figure S10. Temporal decay of community similarity.
