# Supplementary material for: Host Identity and Urban Space Structure Phyllosphere Bacterial Communities Through Compositional Turnover in a Tropical Andean City
Source: Plants (Basel). 2026 Oct 19;15(20):3202. doi: 10.3390/plants15203202 (PMC13708965; doi:10.3390/plants15203202)
Supplement: Supplementary file 1 [file plants-15-03202-s001.zip › Supplementary Tables.pdf]

Table S1. Non-target ASV filtering and taxonomic classification completeness of the bacterial dataset.

Section	Category / taxonomic rank	Number of ASVs	Percentage of initial ASVs (%)	Classified reads (%)	Unclassified reads (%)
**A. Non-target sequence filtering**	Chloroplast / mitochondrial ASVs removed before phylogenetic tree construction	6,362	3.78	—	—
	Chloroplast / mitochondrial ASVs detected in the imported bacterial object	0	0.00	—	—
	Archaea	39	0.02	—	—
	Eukaryota	1	<0.01	—	—
	Unassigned ASVs	53	0.03	—	—
	Bacteria retained for downstream analyses	161,665	96.16	—	—
	**Total initial ASVs**	**168,120**	**100.00**	—	—
**B. Taxonomic classification completeness**	Phylum	—	—	99.44	0.56
	Class	—	—	99.44	0.56
	Order	—	—	99.38	0.62
	Family	—	—	96.92	3.08
	Genus	—	—	84.08	15.92

Table S2. Sequencing depth retained reads and filtered ASVs per sample.

Host plant species	Urban zone	n	Filtered reads, median (min–max)	Filtered ASVs, median (min–max)
Mangifera indica	Center	15	213,878 (74,233–1,043,162)	2,069 (278–3,461)
Mangifera indica	East	15	206,587 (98,756–474,049)	1,273 (116–3,104)
Mangifera indica	North	15	144,375 (80,976–401,211)	1,774 (82–3,514)
Mangifera indica	West	15	175,117 (73,132–334,174)	1,108 (185–2,829)
Mangifera indica	South	15	168,399 (84,773–491,804)	1,136 (67–3,064)
Terminalia catappa	Center	15	186,084 (78,678–375,084)	1,431 (699–3,192)
Terminalia catappa	East	14	160,998 (80,206–401,628)	1,279.5 (40–4,634)
Terminalia catappa	North	15	131,782 (76,590–293,054)	1,392 (80–3,418)
Terminalia catappa	West	15	182,578 (89,515–405,087)	1,614 (77–4,062)
Terminalia catappa	South	15	151,416 (66,221–413,729)	1,648 (43–3,537)
Thunbergia grandiflora	Center	15	150,724 (72,628–668,174)	1,389 (582–2,357)
Thunbergia grandiflora	East	15	140,804 (63,264–441,605)	649 (73–3,977)
Thunbergia grandiflora	North	15	103,359 (49,216–365,192)	1,078 (394–3,831)
Thunbergia grandiflora	West	15	124,057 (78,718–320,168)	1,638 (57–4,599)
Thunbergia grandiflora	South	15	135,408 (79,146–471,812)	1,546 (21–3,382)
**Overall dataset**	—	**224**	**153,048 (49,216–1,043,162)**	**1,460.5 (21–4,634)**

Table S3. Meteorological conditions by urban zone and survey.

Urban zone	Temperature M1 (°C)	Relative humidity M1 (%)	Temperature M2 (°C)	Relative humidity M2 (%)	Temperature M3 (°C)	Relative humidity M3 (%)	Temperature M4 (°C)	Relative humidity M4 (%)	Temperature M5 (°C)	Relative humidity M5 (%)
**Center**	23.0	55.7	23.0	59.4	23.5	63.0	22.0	69.2	21.9	71.7
**East**	22.4	64.8	21.5	73.5	21.9	77.8	21.0	80.9	20.4	93.6
**North**	23.4	57.4	23.4	60.9	23.8	66.3	23.2	68.4	22.3	72.1
**West**	22.7	54.9	22.6	60.2	23.0	63.7	21.9	69.0	21.3	73.2

**South**	24.5	57.1	24.3	61.5	25.2	63.7	23.0	71.6	21.8	79.0

Table S4. Technical diagnostics by survey

A. Survey-level sequencing-depth, ASV exclusivity and dominance diagnostics

Survey	Analyzed subsamples	Filtered reads, min	Filtered reads, median	Filtered reads, max	Survey-exclusive ASVs	Median dominance (%)	Low-richness samples (<100 ASVs)
**M1**	45	173,989	342,529	491,804	4,667	6.2	0
**M2**	45	118,678	202,202	268,978	499	12.7	0
**M3**	45	76,760	142,653	232,190	492	7.3	0
**M4**	44	49,216	105,891	1,043,162	66	23.4	12
**M5**	45	65,933	105,834	474,049	202	15.8	0

B. Low-richness samples identified in survey M4

Sample ID	Survey	Host plant species	Urban zone	Observed ASVs	Dominant taxon	Dominance (%)
**04ST2**	M4	*Thunbergia grandiflora*	South	20	*Serratia*	53.7
**04EA1**	M4	*Terminalia catappa*	East	40	Unresolved	54.7
**04SA5**	M4	*Terminalia catappa*	South	40	Unresolved	49.5
**04OT4**	M4	*Thunbergia grandiflora*	West	50	*Pantoea*	96.4
**04SA1**	M4	*Terminalia catappa*	South	64	*Serratia*	45.6
**04SM6**	M4	*Mangifera indica*	South	66	*Pantoea*	41.6
**04ET3**	M4	*Thunbergia grandiflora*	East	71	*Serratia*	44.6
**04OA4**	M4	*Terminalia catappa*	West	73	Unresolved	18.2
**04ET2**	M4	*Thunbergia grandiflora*	East	74	Unresolved	49.5
**04SM2**	M4	*Mangifera indica*	South	77	*Kosakonia*	22.9
**04NM1**	M4	*Mangifera indica*	North	78	Unresolved	29.4
**04NA5**	M4	*Terminalia catappa*	North	79	*Enterobacter*	30.5

Table S5. Relationship between sequencing depth and Bray–Curtis ordination axes before and after rarefaction.

PCoA axis	Spearman's ρ before rarefaction	*p*-value	Spearman's ρ after rarefaction	*p*-value
**PCoA1**	−0.163	0.014	−0.153	0.023
**PCoA2**	−0.427	<0.001	−0.427	<0.001

Table S6. Global taxonomic composition of epiphytic phyllosphere bacterial communities and survey-specific abundance of Enterobacteriaceae.

A. Dominant bacterial taxa in the ecology-filtered dataset

Taxonomic rank	Taxon	Relative abundance (%)
**Phylum**	Proteobacteria	62.60
**Phylum**	Actinobacteriota	13.68
**Phylum**	Bacteroidota	6.98
**Phylum**	Deinococcota	5.96
**Phylum**	Firmicutes	4.03
**Phylum**	Cyanobacteria	2.01
**Phylum**	Myxococcota	1.12
**Phylum**	Chloroflexi	0.79
**Phylum**	Unclassified	0.56
**Phylum**	Acidobacteriota	0.54
**Family**	Beijerinckiaceae	14.12

**Family**	Erwiniaceae	12.02
**Family**	Enterobacteriaceae	11.35
**Family**	Deinococcaceae	5.66
**Family**	Acetobacteraceae	5.43
**Family**	Hymenobacteraceae	4.65
**Family**	Sphingomonadaceae	3.99
**Family**	Microbacteriaceae	3.12
**Family**	Oxalobacteraceae	2.78
**Family**	Yersiniaceae	2.52
**Family**	Pseudomonadaceae	2.12
**Family**	Unclassified Enterobacterales	1.85
**Genus**	*Pantoea*	10.19
**Genus**	*Methylobacterium–Methylorubrum*	10.00
**Genus**	*Deinococcus*	5.66
**Genus**	Unclassified Enterobacteriaceae	5.02
**Genus**	*Hymenobacter*	4.62
**Genus**	*Sphingomonas*	3.20
**Genus**	1174-901-12	3.04
**Genus**	*Enterobacter*	2.92
**Genus**	Unclassified Bacteria	2.71
**Genus**	*Massilia*	2.68
**Genus**	*Serratia*	2.52
**Genus**	Unclassified Acetobacteraceae	2.46
**Genus**	*Pseudomonas*	2.12
**Genus**	*Craurococcus–Caldovatus*	1.60
**Genus**	*Curtobacterium*	1.41

B. Relative abundance of Enterobacteriaceae by survey

Survey	Median relative abundance (%)	Mean relative abundance (%)
**M1**	0.04	1.11
**M2**	0.70	10.21
**M3**	0.29	0.88
**M4**	14.69	36.24
**M5**	0.81	8.87

Table S7. Plant-level alpha-diversity tests for host species and urban zone (n = 15).

Alpha-diversity metric	Host species F (df)	*p*-value	Urban zone F (df)	*p*-value
**Observed ASVs**	0.18 (2, 8)	0.837	0.90 (4, 8)	0.508
**Chao1 richness**	0.18 (2, 8)	0.838	0.76 (4, 8)	0.579
**Shannon diversity**	0.57 (2, 8)	0.585	2.70 (4, 8)	0.108
**Simpson diversity**	0.39 (2, 8)	0.689	3.78 (4, 8)	0.052
**Inverse Simpson diversity**	2.21 (2, 8)	0.172	0.58 (4, 8)	0.686
**Faith's phylogenetic diversity**	0.40 (2, 8)	0.681	3.06 (4, 8)	0.083

Table S8. Post hoc pairwise contrasts among surveys for alpha-diversity metrics.

Survey contrast	Observed ASVs	Chao1	Shannon	Simpson	Inverse Simpson	Faith's PD
**M1 – M2**	1083.89 (<0.001)	1294.58 (<0.001)	1.044 (0.001)	0.060 (0.032)	57.16 (0.015)	34.53 (<0.001)
**M1 – M3**	1193.09 (<0.001)	1450.61 (<0.001)	0.667 (0.033)	0.019 (0.510)	27.13 (0.216)	41.86 (<0.001)
**M1 – M4**	2288.80 (<0.001)	2579.27 (<0.001)	3.021 (<0.001)	0.164 (<0.001)	106.74 (<0.001)	104.32 (<0.001)
**M1 – M5**	1954.31 (<0.001)	2238.76 (<0.001)	1.914 (<0.001)	0.070 (0.014)	97.03 (<0.001)	76.11 (<0.001)
**M2 – M3**	109.20 (0.490)	156.03 (0.363)	−0.378 (0.210)	−0.041 (0.127)	−30.03 (0.190)	7.33 (0.309)
**M2 – M4**	1204.91 (<0.001)	1284.70 (<0.001)	1.976 (<0.001)	0.104 (<0.001)	49.58 (0.033)	69.79 (<0.001)
**M2 – M5**	870.42 (<0.001)	944.18 (<0.001)	0.869 (0.006)	0.010 (0.697)	39.87 (0.084)	41.58 (<0.001)
**M3 – M4**	1095.71 (<0.001)	1128.66 (<0.001)	2.354 (<0.001)	0.145 (<0.001)	79.61 (0.001)	62.46 (<0.001)
**M3 – M5**	761.22 (<0.001)	788.15 (<0.001)	1.247 (<0.001)	0.051 (0.064)	69.90 (0.003)	34.25 (<0.001)
**M4 – M5**	−334.49 (0.042)	−340.51 (0.056)	−1.107 (<0.001)	−0.094 (<0.001)	−9.72 (0.640)	−28.21 (<0.001)

Table S9. Tests for homogeneity of multivariate dispersion across host species, urban zones, and surveys.

Analytical level / beta-diversity metric	Host species, F (*p*)	Urban zone, F (*p*)	Survey, F (*p*)
**Plant-level profiles (*n* = 15)**	0.16 (0.859)	0.36 (0.826)	—
**Bray–Curtis**	0.09 (0.915)	2.45 (0.047)	32.51 (<0.001)
**Jaccard**	0.15 (0.864)	3.56 (0.009)	36.37 (<0.001)
**Aitchison/CLR**	0.63 (0.529)	0.42 (0.787)	97.16 (<0.001)
**Weighted UniFrac**	0.12 (0.890)	1.75 (0.132)	8.61 (<0.001)
**Unweighted UniFrac**	0.36 (0.702)	3.05 (0.017)	27.80 (<0.001)

Table S10. Bray–Curtis dissimilarity across temporal intervals between surveys.

Temporal interval (days)	Pairwise comparisons (n)	Median Bray–Curtis dissimilarity	Mean Bray–Curtis dissimilarity
**71**	135	0.605	0.611
**131**	132	0.890	0.833
**132**	135	0.676	0.680
**203**	135	0.632	0.651
**231**	132	0.932	0.880
**362**	135	0.789	0.770
**363**	132	0.932	0.847
**434**	132	0.946	0.863
**494**	135	0.757	0.736
**565**	135	0.732	0.726

Table S11. Within-plant heterogeneity and null-model controls of plant-level community coherence

Grouping factor	Level	Within-plant pairs (n)	Median within-plant Bray–Curtis	Mean within-plant Bray–Curtis	Median between-plant Bray–Curtis	Within/between ratio	Null expectation, mean (95% interval)	SES	*p*-value
**Host species**	*Mangifera indica*	75	0.539	0.590	—	—	—	—	—
**Host species**	*Terminalia catappa*	73	0.535	0.569	—	—	—	—	—
**Host species**	*Thunbergia grandiflora*	75	0.574	0.593	—	—	—	—	—
**Survey**	M1	45	0.434	—	0.702	0.617	0.698 (0.668–0.727)	−17.26	0.001
**Survey**	M2	45	0.494	—	0.782	0.631	0.776 (0.735–0.808)	−16.45	0.001
**Survey**	M3	45	0.501	—	0.771	0.651	0.765 (0.730–0.795)	−14.66	0.001
**Survey**	M4	43	0.907	—	0.964	0.941	0.961 (0.937–0.977)	−5.48	0.001
**Survey**	M5	45	0.680	—	0.853	0.798	0.848 (0.814–0.878)	−10.29	0.001

Table S12. Sensitivity analyses and screening of potential contaminant-associated genera.

A. Sensitivity analyses after exclusion of the 12 low-richness M4 samples

Analysis	Metric	Factor	F / R^2	*p*-value	n
**Alpha diversity**	Observed ASVs	Host species	F = 0.76	0.475	212
		Urban zone	F = 1.63	0.187	212
		Survey	F = 104.48	<0.001	212
	Shannon	Host species	F = 1.11	0.333	212
		Urban zone	F = 3.20	0.014	212
		Survey	F = 34.19	<0.001	212
	Simpson	Host species	F = 0.64	0.530	212
		Urban zone	F = 3.86	0.005	212
		Survey	F = 8.37	<0.001	212
	Faith's PD	Host species	F = 1.55	0.216	212
		Urban zone	F = 4.77	0.001	212
		Survey	F = 88.30	<0.001	212
**Beta diversity**	Bray–Curtis PERMANOVA	Host species	R^2 = 0.096	<0.001	212
		Urban zone	R^2 = 0.095	<0.001	212
		Survey	R^2 = 0.092	<0.001	212

B. Genera included in the potential-contaminant screening

Genus	Number of ASVs	Mean relative abundance (%)

Enterobacter	24	2.920
Serratia	25	2.522
Variovorax	13	0.151
Staphylococcus	28	0.083
Stenotrophomonas	32	0.041
Escherichia–Shigella	16	0.027
Bradyrhizobium	3	0.022
Delftia	3	0.005
Acidovorax	16	0.003
Cutibacterium	1	0.001
Ralstonia	1	<0.001
Pelomonas	4	<0.001
Achromobacter	3	<0.001
